# Alterations in stress granule dynamics driven by TDP-43 and FUS: a link to pathological inclusions in ALS?

**DOI:** 10.3389/fncel.2015.00423

**Published:** 2015-10-23

**Authors:** Anaïs Aulas, Christine Vande Velde

**Affiliations:** ^1^Centre de Recherche du Centre Hospitalier de l’Université de MontréalMontréal, QC, Canada; ^2^Department of Biochemistry, Université de MontréalMontréal, QC, Canada; ^3^Department of Neurosciences, Université de MontréalMontréal, QC, Canada

**Keywords:** TDP-43, FUS, stress granules, microtubules, pathological inclusions, amyotrophic lateral sclerosis, frontotemporal dementia

## Abstract

Stress granules (SGs) are RNA-containing cytoplasmic foci formed in response to stress exposure. Since their discovery in 1999, over 120 proteins have been described to be localized to these structures (in 154 publications). Most of these components are RNA binding proteins (RBPs) or are involved in RNA metabolism and translation. SGs have been linked to several pathologies including inflammatory diseases, cancer, viral infection, and neurodegenerative diseases such as amyotrophic lateral sclerosis (ALS) and frontotemporal dementia (FTD). In ALS and FTD, the majority of cases have no known etiology and exposure to external stress is frequently proposed as a contributor to either disease initiation or the rate of disease progression. Of note, both ALS and FTD are characterized by pathological inclusions, where some well-known SG markers localize with the ALS related proteins TDP-43 and FUS. We propose that TDP-43 and FUS serve as an interface between genetic susceptibility and environmental stress exposure in disease pathogenesis. Here, we will discuss the role of TDP-43 and FUS in SG dynamics and how disease-linked mutations affect this process.

## Stress Granules: Generalities

**FIGURE 1:**
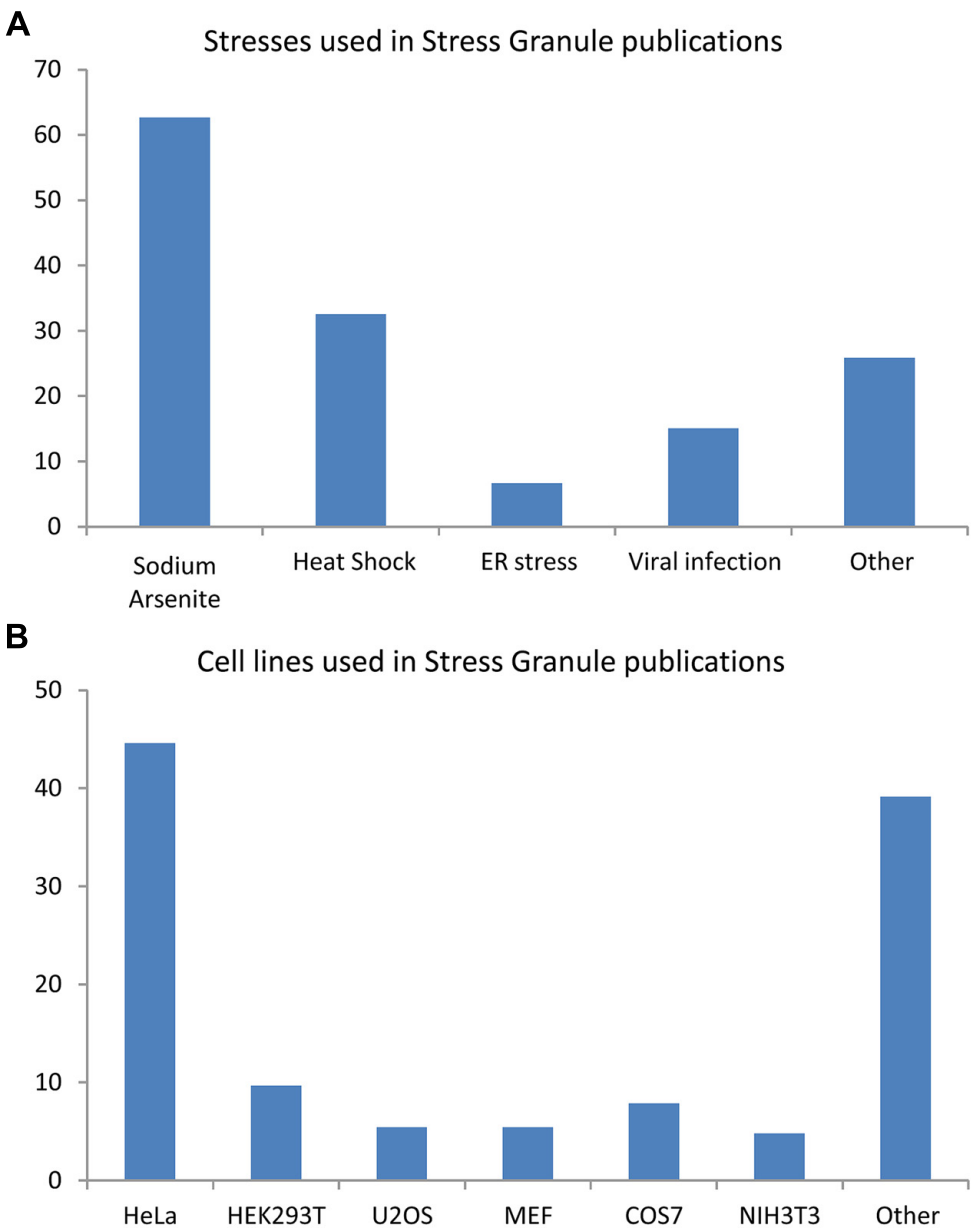
**Cell lines and stress inducing agents used in stress granule studies.** Analysis of 154 articles published between 1999 and 2014. Some publications used many cells type and/or many stresses. **(A)** Stresses involved in stress granule (SG) studies. **(B)** Cell lines used in SG studies.

Stress granule (SG) formation is a pan-cellular mechanism employed to counter exposures to osmotic (Goulet et al., 2008; Dewey et al., 2011), oxidative (Kedersha et al., 1999; Stohr et al., 2006; McDonald et al., 2011), mitochondrial (Stoecklin et al., 2004; Chalupnikova et al., 2008), or endoplasmic reticulum (ER) stress (Kimball et al., 2003; Goodier et al., 2007), viral infection (Emara and Brinton, 2007; Raaben et al., 2007), proteasome inhibition (Mazroui et al., 2007; Colombrita et al., 2009; Fournier et al., 2010), inhibition of translation initiation (Dang et al., 2006; Mazroui et al., 2006), ultraviolet light (Kwon et al., 2007; Pothof et al., 2009), cadmium chloride (Bravard et al., 2010) as well as certain anti-cancer (Leung et al., 2006b; Mazroui et al., 2006; Fujimura et al., 2012) and antifungal drugs (Ohn et al., 2008; Bentmann et al., 2012), (**Figure 1A**). An analysis of 154 reports published between 1999 and 2014 reveals that the majority of our knowledge of these cytoplasmic foci derives from studies in HeLa cells (45%) using sodium arsenite (SA) as a means of oxidative stress (63%) or thermal stress (33%; **Figure 1B**). It is noteworthy that not all environmental conditions induce a SG response. Namely, inhibition of RNA polymerase, exposure to inflammatory cytokines, nutrient depletion, and destabilization of microtubules and/or actin microfilaments do not induce SG formation in mammalian cells (Kedersha et al., 1999).

Stress granules were first defined as cytoplasmic foci containing polyadenylated RNA, small ribosomal sub-units, translation initiation factors (eIF3, eIF4E, eIF4G), and RNA binding proteins (RBPs) such as TIA-1, HuR, PABP, and TTP that formed following eIF2α phosphorylation. Furthermore, SGs formed from numerous small inclusions that fused over time and dissolved once the stress abated, and were inhibited by cycloheximide treatment (Kedersha et al., 1999). This definition still applies today, but the number and variety of proteins reported as recruited to SGs has exploded.

Stress granules are distinct from other RNA granule inclusions called Processing Bodies (PBs), even though they share some common protein components under certain conditions (Kedersha et al., 2005; Dang et al., 2006; Leung et al., 2006a; Wasserman et al., 2010). PBs do not contain initiation elongation factors, with the exception of some eIF4 subunits (Kedersha et al., 2005; Teixeira et al., 2005). PBs contain proteins involved in translational repression and mRNA degradation (Parker and Sheth, 2007; Kulkarni et al., 2010) and are present in basal conditions but can also be induced by some stress exposures (Sheth and Parker, 2003; Kedersha et al., 2005). For example, SA induces both structures while heat shock or clotrimazole induce only SGs (Kedersha et al., 2005). PB formation is dependent on available mRNA in the cytoplasm (Kedersha et al., 2005; Teixeira et al., 2005; Parker and Sheth, 2007), and is independent of eIF2α phosphorylation (Kedersha et al., 2005). SGs and PBs can also interact in a process referred to as “docking” (Kedersha et al., 2005; Parker and Sheth, 2007; Aulas et al., 2015). How SGs and PBs function together in the stress response is expected to be at the center of future important discoveries.

### Protein Composition of Stress Granules

**FIGURE 2:**
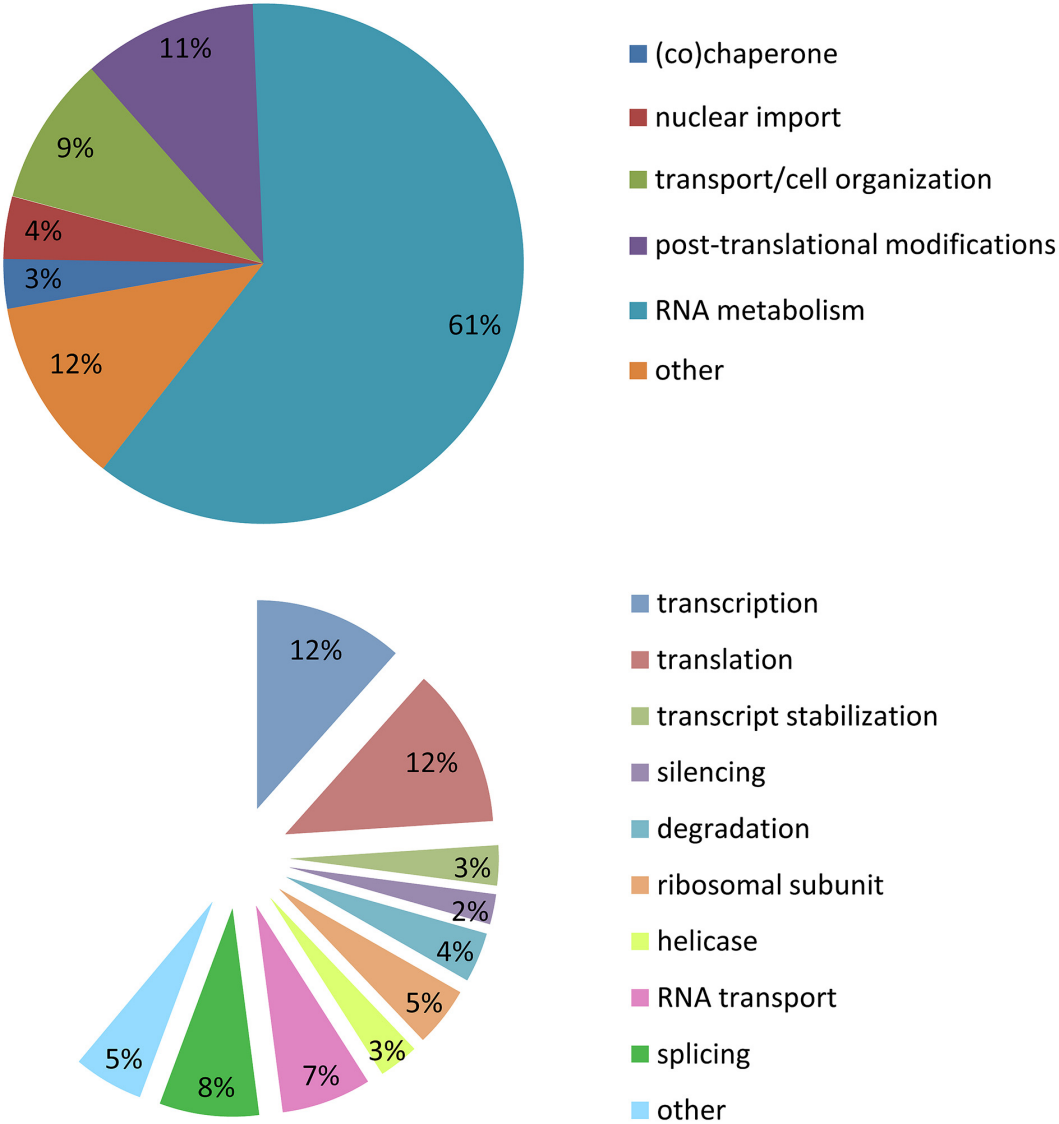
**Stress granule composition.** Analysis of 154 publications between 1999 and 2014. **(A)** Cellular metabolism functions of proteins recruited to SGs. **(B)** Specific function in mRNA metabolism of proteins recruited to SGs.

Stress granules are extremely labile inclusions, non-limited by membranes (Nover et al., 1989; Kedersha et al., 2000, 2005; Kedersha and Anderson, 2002; Bosco et al., 2010). Many of the RBPs found in SGs contain low complexity domains which are prone to aggregation. These multimeric protein assemblies are neither soluble nor insoluble but proposed to be in an intermediate labile state referred to as a “hydrogel”. This unique feature permits SGs to be highly dynamic and in constant exchange with cytoplasmic components (Han et al., 2012; Kato et al., 2012). It is for this reason that there is no published biochemical method to purify SGs so that their composition and function may be more deeply interrogated. In our analysis of SG-related publications (1999–2014), the majority of the proteins described as localized to SGs belong to the large family of RBPs (60%). A similar proportion are implicated in RNA metabolism including transcription (12%), splicing (8%), RNA transport (7%), degradation (4%), silencing (2%), mRNA stabilization (3%), and translation (12%). There are also helicases (4%) and small ribosomal proteins (5%). Of the remaining 40% of SG-recruited proteins not involved in RNA metabolism, 11% have a role in post-translational modification, 9% participate in cell organization/protein transport, 4% in nuclear import, 3% are (co)-chaperones, and 13% are sufficiently diverse to be classified as other (**Figure 2**; Supplementary Table S1). These descriptors have led to the proposal that SGs might also serve to integrate complex cellular signaling (Kedersha et al., 2013).

Stress granules contain translation initiation factors eIF3, eIF4, eIF5 (Elongation Initiation Factor 3–5; Kedersha et al., 2002; Li et al., 2010), small ribosomal subunits (S3, S6, S18, S19; Kedersha et al., 2002; Kimball et al., 2003; Farny et al., 2009), and numerous RBPs. Among the latter group, TIA-1 (T-cell-restricted intracellular antigen-1) and G3BP1 (Ras GAP SH3 domain-binding protein 1) are the two most commonly studied and utilized SG markers (Kedersha et al., 1999; Tourriere et al., 2003; Gilks et al., 2004). The overexpression of either of these proteins is sufficient to drive the formation of cytoplasmic inclusions, often referred to as “spontaneous” or “constitutive” SGs (Tourriere et al., 2003; Gilks et al., 2004; Reineke et al., 2012). Both TIA-1 and G3BP1 feature a glycine-rich domain, also known as a prion-like, low complexity or intrinsically disordered domain, which facilitates the first step of SG formation (Kedersha and Anderson, 2002; Tourriere et al., 2003; Gilks et al., 2004). Historically, TIA-1 was the most frequently studied marker of SGs, but has now been surpassed by G3BP1 in the literature.

G3BP1 was first described as a nuclear mRNA binding protein preferentially expressed in the brain (Parker et al., 1996; Martin et al., 2013). It is implicated in mRNA degradation via its endoribonuclease activity induced by its phosphorylation at Ser^149^ (Gallouzi et al., 1998; Tourriere et al., 2001). Coincidentally, Ser^149^ phosphorylation inhibits SG formation (Tourriere et al., 2003). That G3BP1 is central to SG dynamics is supported by the observation that G3BP1 function is often circumvented during viral infections. Most strikingly, during poliovirus infection, G3BP1 is cleaved by the viral protease 3C. Consequently, infected cells exposed to heat shock have disrupted SG dynamics such that SG size is diminished compared to non-infected heat-shocked cells (White et al., 2007; Piotrowska et al., 2010). In primary embryonic fibroblasts derived from these G3BP1^-/-^ mice, when SGs are able to form, they are less numerous, smaller and less well-defined (Jedrusik-Bode et al., 2013). It is noteworthy to mention that G3BP1 is a neuronal survival factor since G3BP1-null mice die in the neonatal phase owing to wide-spread neuronal cell death within the central nervous system within 15 min of being *ex utero* (Zekri et al., 2005). A second G3BP1-null model was created that generates viable pups but demonstrates clearly that G3BP1 is critical for synaptic plasticity and calcium homeostasis, establishing a link between SGs and neurodegeneration (Martin et al., 2013). Taken together, these data indicate that G3BP1 is an essential component regulating SG dynamics and is relevant to neurons. G3BP2, a close homolog of G3BP1, is also recruited to SGs and has been suggested to partially compensate for the loss of G3BP1 (Kobayashi et al., 2012; Matsuki et al., 2013). However, it has been recently demonstrated that while G3BP1 is necessary for SG secondary aggregation and SG function, G3BP2 is dispensable for these aspects, although simultaneous down-regulation of both abrogates SG formation (Aulas et al., 2015). Further studies are needed to clearly define the role of G3BP2 in SGs.

### Stress Response in Neurodegenerative Diseases

Neurodegenerative diseases are often characterized by pathological inclusions, a subset of which colocalize with SG markers. For example, TIA-1 co-localizes with neuronal inclusions formed in response to expression of the first exon of the *Huntingtin* gene containing a polyglutamine expansion (a well described model for Huntington disease; Waelter et al., 2001). In two different mouse models of Alzheimer’s disease expressing Tau, three different cytoplasmic inclusions containing TIA-1, G3BP1, or TTP (Tristetraprolin, an RBP recruited to SGs) are observed. The presence and the size of all of those inclusions correlate with disease severity (Vanderweyde et al., 2012). TIA-1 and TTP colocalize with phosphorylated Tau inclusions in murine models and in post-mortem patient tissues primarily at later disease stages. In contrast, G3BP1 inclusions have weak immunoreactivity for pathological Tau (marked by PHF-1 antibody) at any stage. Interestingly, although TDP-43 (TAR DNA binding protein 43) cytoplasmic inclusions were also observed in animals with moderate to severe pathology, they did not co-label with TIA-1 or PHF-1 (Vanderweyde et al., 2012).

In contrast, in Amyotrophic Lateral Sclerosis (ALS) and Frontotemporal Dementia (FTD), subsets of TDP-43 containing cytoplasmic inclusions label for SG markers such as TIA-1, eIF3 and PABP (Polyadenylate-binding protein; Volkening et al., 2009; Dormann et al., 2010; Liu-Yesucevitz et al., 2010; Bentmann et al., 2012; McGurk et al., 2014), (**Table 1**). It has been proposed that the formation of these pathological inclusions may be due to misregulation of the SG response. Indeed, one of the most prevalent hypotheses is that inclusion formation is driven by the failure of SGs to disassemble. At present, there is no data nor methodology available to determine if these inclusions are broadly composed of SG proteins or if SG proteins are themselves recruited to pre-formed inclusions (Bentmann et al., 2013).

**Table 1:** Stress granule markers observed in pathological amyotrophic lateral sclerosis/frontotemporal dementia (ALS/FTD) inclusions.

ALS protein	SG markers		Processing Bodies (PB) markers	Patients	Tissue	Reference


	Positive	Negative				
TDP-43		TIA-1, HuR		3 Sporadic ALS	Spinal cord	Colombrita et al., 2009
TDP-43	TIA-1			Sporadic ALS		Volkening et al., 2009
			XRN1, not frequent			
FUS	PABP1, eIF4G			1 Familial ALS-FUS^R521C^	Spinal cord	Dormann et al., 2010
				7 Sporadic FTD-FUS	Hippocampus	
TDP-43		PABP1, eIF4G		2 Sporadic FTD-TDP		
TDP-43	eIF3, TIA-1		Negative for Dcp1A (not shown)	ALS FTD	Spinal cord Frontal cortex	Liu-Yesucevitz et al., 2010
TDP-43	PABP1 (66% of cases)			ALS-TDP	Spinal cord	Bentmann et al., 2012
		PABP1		FTD-TDP	Hippocampus	

### Pathological Inclusions in ALS/FTD

Ubiquitin-positive inclusions are primarily observed in neurons and sometimes glial cells of ALS and FTD post-mortem tissues from the central nervous system (Arai et al., 2006; Neumann et al., 2006; Ling et al., 2013). They typically contain one of two RBPs known to also harbor disease-causing mutations, TDP-43 or FUS (Fused in liposarcoma; Colombrita et al., 2009; Volkening et al., 2009; Dormann et al., 2010; Liu-Yesucevitz et al., 2010; Bentmann et al., 2012). TDP-43 is a major resident of these pathological inclusions being detected in 97% of all ALS cases and 45% for FTD. In contrast, FUS-immunoreactive inclusions are found in only 2% of ALS and 9% of FTD cases (Arai et al., 2006; Neumann et al., 2006; Ling et al., 2013). The functional significance of these structures remains poorly defined and several possibilities have arisen. First, inclusions could be indirectly harmful due to inappropriate sequestration of critical cellular signaling proteins. Second, the pathological process or mutations could induce protein misfolding, so as to affect cell signaling and enhance cell vulnerability. On the other hand, inclusions could also be considered as neuroprotective due to sequestration of misfolded protein. Lastly, inclusions could be inert and have no direct link to or bearing on disease pathogenesis. Which of these scenarios is correct is still not yet understood and a major focus of the field (Arai et al., 2006; Neumann et al., 2006; Kabashi et al., 2008; Sreedharan et al., 2008; Kwiatkowski et al., 2009; Vance et al., 2009; Ling et al., 2013).

### From Inclusions to Stress Granules?

The presence of SG markers within TDP-43 positive inclusions has led to the hypothesis that pathological TDP-43/FUS containing inclusions originate from SGs that have failed to disassemble. While TDP-43 and FUS inclusions have been reported to clearly co-localize with some specific SG markers, it is not a universal finding for both proteins (Colombrita et al., 2009; Volkening et al., 2009; Dormann et al., 2010; Liu-Yesucevitz et al., 2010; Bentmann et al., 2012), (**Table 1**). In addition, these studies should perhaps be interpreted with some measure of caution since some of the SG markers used in these studies, including TIA-1 and HuR, are reported to label other RNA granule subtypes (such as PBs) in certain contexts (Dang et al., 2006; Leung et al., 2006b). In our view, the question remains very much open as to whether inclusions derive from SGs.

**FIGURE 3:**
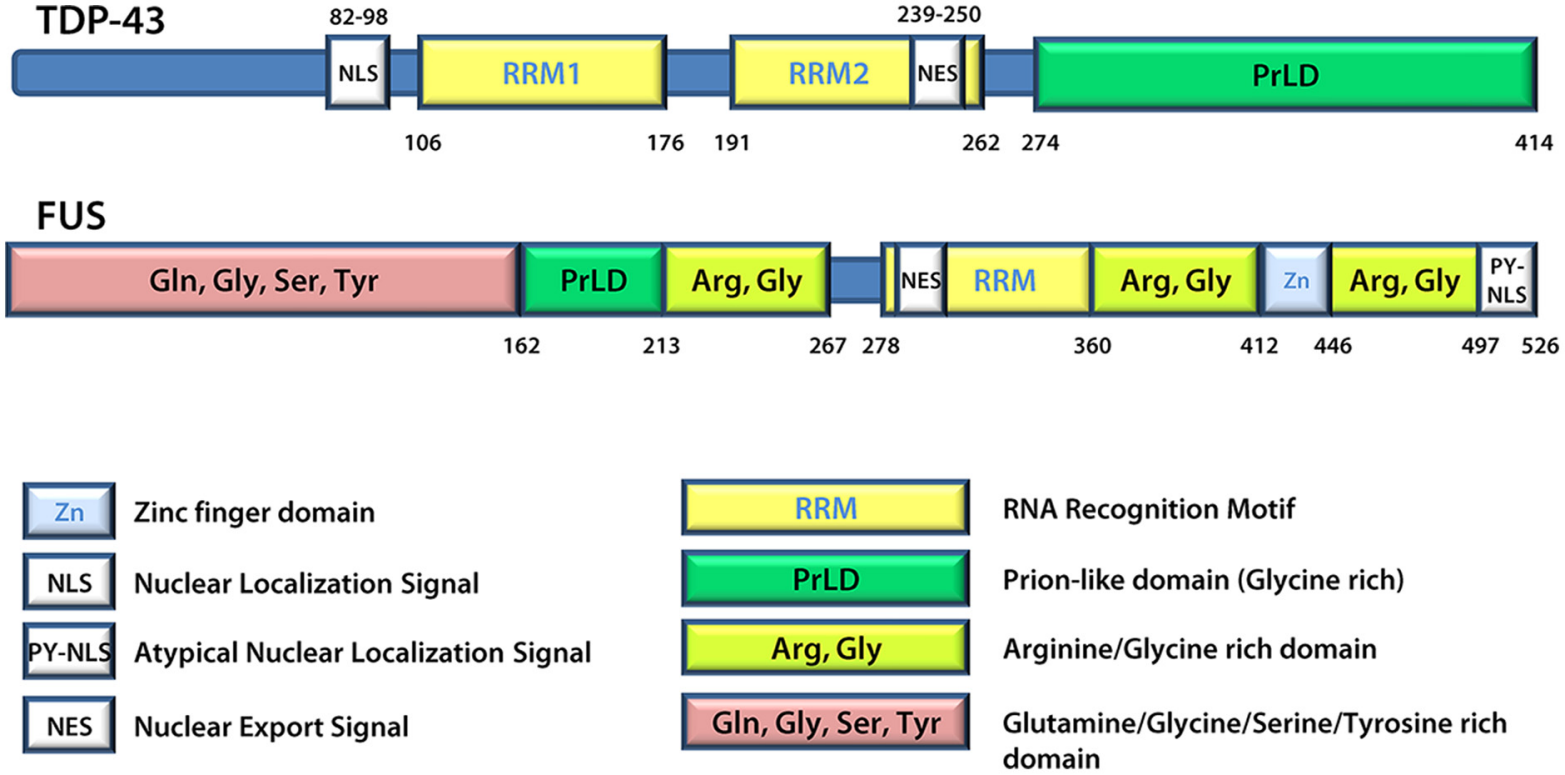
**TDP-43 and FUS protein structures.** FUS and TDP-43 have structural similarities with both harboring a Prion-like domain, RNA recognition motif(s), nuclear localization signal, and nuclear export signal. Details regarding the position of domains were derived from UniProt.

A number of proteins linked to ALS are found in SGs and/or patient inclusions leading to the proposal that mutant disease proteins may interfere in the normal SG response during pathogenesis. These include VCP (Valosin-containing protein; Abramzon et al., 2012; Buchan et al., 2013; Seguin et al., 2014), SMN (Survival of motor neuron; Hua and Zhou, 2004; Piao et al., 2011), hnRNP A1 and hnRNP A2 (Heterogeneous nuclear ribonucleoprotein A1 and A2; McDonald et al., 2011; Kim et al., 2013), TAF15 (TATA-binding protein-associated factor 15; Andersson et al., 2008; Couthouis et al., 2011), Angiogenin (Greenway et al., 2004; Conforti et al., 2008; Emara et al., 2010) and Profilin1 (Wu et al., 2012; Figley et al., 2014). However, generally the focus is on TDP-43 and FUS (Andersson et al., 2008; Colombrita et al., 2009; Bosco et al., 2010; Freibaum et al., 2010; Liu-Yesucevitz et al., 2010; Dewey et al., 2011; McDonald et al., 2011; Bentmann et al., 2012). TDP-43 and FUS share many structural similarities (**Figure 3**) and both are involved in various aspects of mRNA metabolism including splicing, nucleocytoplasmic shuttling, transcription, mRNA stability, and SG dynamics (Lagier-Tourenne et al., 2010). Specifically, they both have prion-like domains (Cushman et al., 2010; King et al., 2012; Li et al., 2013), and the combination of a prion-like domain and a RRM has recently been used to predict genetic modifiers or causes of several neurodegenerative diseases (King et al., 2012). Thus, it has been proposed that these two proteins may participate in a common pathogenic mechanism. In ALS, exposure to external stress is frequently proposed as a contributor to either disease initiation or rate of progression (D’Amico et al., 2013). Given that TDP-43 and FUS are both recruited to SGs and modulate the SG response (see below), it is reasonable to propose that they may serve as an interface between genetic susceptibility and environmental stress exposure in disease pathogenesis. This thus explains the intensifying interest around these molecules and their involvement in the SG response.

## Localization Of TDP-43 and FUS to Stress Granules

Between 2010 and 2012, five different teams evaluated the link between TDP-43 and SGs. All used different cell lines, different stress-inducing agents, and different SG markers. From this collective work, it is now recognized that TDP-43 is recruited to SGs following a range of stress stimuli including ER, osmotic, oxidative, thermal, and mitochondrial stress as well as proteasome inhibition. While all of these studies used different markers, there is consensus that TDP-43 is recruited uniquely to SGs and not PBs (**Table 2**). Localization of TDP-43 to SGs is mediated by both its RRM1 domain as well as its C-terminal glycine-rich/prion-like domain (Colombrita et al., 2009; Dewey et al., 2011). These data imply that the binding of TDP-43 to mRNA via its RRM1 as well as proteins, via the C-terminus, is relevant to its SG localization. Using transient overexpression of TDP-43, only one team has reported that exogenous TDP-43 induces the formation of “constitutive SGs” (Liu-Yesucevitz et al., 2010). These results are reminiscent of what is observed following high-level overexpression of TIA-1 and G3BP1 and thus may reflect the consequence of supra-expression of aggregation-prone proteins which feature a prion-like domain (Kedersha et al., 1999; Tourriere et al., 2003). Moreover, other groups do not observe these TDP-43 induced structures (Colombrita et al., 2009; Dewey et al., 2011; Walker et al., 2013). While this discrepancy remains to be resolved, whether cytoplasmic aggregates induced by TDP-43, TIA-1, or G3BP1 overexpression satisfy the consensus definition of SGs also remains to be determined.

**Table 2:** TDP-43 localization to SGs.

Cell type		Stress	SG markers	TDP-43 visualization	Reference
HEK293T		0.4 M sorbitol, 1 h	HuR, hnRNPA1, TIAR	Overexpression + Endogenous	Dewey et al., 2011
Cortical glia					
BE-M17	HEK293	0.5 mM SA, 1 h	TIA-1, TIAR, PABP, eIF3	Overexpression	Liu-Yesucevitz et al., 2010
		Hanks balanced salt solution			
NSC34		0.5 mM SA, 30 min	TIA-1, HuR	Overexpression + Endogenous	Colombrita et al., 2009
		Heat shock, 44°C, 30 min			
		10 μM MG132, 4 h			
SK-N-SH		0.5 mM SA, 30 min	TIA-1	Endogenous	McDonald et al., 2011
HeLa		0.5 mM SA, 30 min			
		Heat shock, 43°C, 30 min			
		1 μM thapsigargin, 50 min			

Exogenously expressed mutant FUS is recruited to SGs in response to oxidative, thermal, mitochondrial and ER stress (Andersson et al., 2008; Bosco et al., 2010; Bentmann et al., 2012; Daigle et al., 2013), (**Table 3**). Interestingly, while GFP-FUS^WT^ is recruited to less than 10% of TIAR-positive SGs (Andersson et al., 2008; Bentmann et al., 2012), SG localization is exacerbated when FUS is mutated (Bosco et al., 2010; Baron et al., 2013; Lenzi et al., 2015). In contrast, endogenous FUS is reported to robustly localize to SGs only in conditions of hyperosmolarity (Sama et al., 2013) and does not colocalize with the PB marker GE-1/HEDLS (Bosco et al., 2010). Localization of FUS to SGs is independent of its glycine-rich/prion-like domain but does require its capacity to bind mRNA (Andersson et al., 2008; Bentmann et al., 2012; Daigle et al., 2013). Mutations in FUS frequently disrupt the nuclear localization sequence (NLS), thereby leading to an accumulation of FUS in the cytoplasm that contributes to its increased recruitment to SGs (Vance et al., 2013). Intriguingly, mutant FUS localized to SGs will further recruit wild type FUS protein (Vance et al., 2013). Lastly, overexpression of FUS (wild type or mutant) itself does not induce “constitutive SGs” but rather requires a stress stimulus to trigger SG localization (Bosco et al., 2010; Daigle et al., 2013).

**Table 3:** FUS localization to SGs.

Cell types		Stress conditions	SG markers	FUS visualization	Reference
HeLa	HT-1080	0.5 mM SA, 1 h	TIA-1	Endogenous and overexpressed wild type protein	Andersson et al., 2008
		Heat shock, 44°C, 1h			
SH-SY5Y	primary hippocampal neurons	Heat shock, 44°C, 1h	TIA-1	Overexpression of mutant proteins	Bentmann et al., 2012
		0.5 mM SA, 30 min			
		20 μM clotrimazole, 30 min			
HEK293		0.5 mM SA, 1 h	TIAR	Overexpression of mutant and wild type proteins	Bosco et al., 2010
		10 μM thapsigargin, 2 h			
		Heat shock 42.5°C, 30 min			
fish embryos		Heat shock 42.5°C, 45 min			
HeLa		0.4 M sorbitol, 20 min or 1 h	G3BP, TIAR	Endogenous wild type protein	Sama et al., 2013

### The Role of Endogenous TDP-43 and FUS in Stress Granule Dynamics

While many teams have analyzed the effect of mutant or wild type proteins on SG phenotypes using overexpression in a range of transformed cell lines (Liu-Yesucevitz et al., 2010; Dewey et al., 2011; Baron et al., 2013; Walker et al., 2013), there are only a handful that have examined the role of the endogenous proteins in SG dynamics (Colombrita et al., 2009; McDonald et al., 2011; Aulas et al., 2012, 2015; Blechingberg et al., 2012). Importantly, decreasing the expression of either TDP-43 or FUS does not abolish SG formation (Colombrita et al., 2009; Liu-Yesucevitz et al., 2010; McDonald et al., 2011; Aulas et al., 2012; Blechingberg et al., 2012; Sama et al., 2013). In response to arsenite-induced oxidative stress, TDP-43 depletion does not influence eIF2α phosphorylation (McDonald et al., 2011). However, SG dynamics are affected at several levels such that SG assembly is delayed, secondary aggregation is abrogated and disassembly is accelerated (McDonald et al., 2011). The SG proteins G3BP1 and TIA-1 are down and up-regulated, respectively, in cells depleted of TDP-43 (McDonald et al., 2011). In addition, at early time points, SGs have a diffuse and more irregular morphology (McDonald et al., 2011). This latter observation prompted a deeper investigation of how TDP-43 influences the assembly process. Normally, SGs form initially as multiple small cytoplasmic puncta, which gradually coalesce into larger and less numerous structures. This process, sometimes referred to as secondary aggregation, is completely abolished when TDP-43 levels are reduced (Aulas et al., 2012). The significance of this two-step assembly of SGs has been enigmatic. However, it has recently been uncovered that SG coalescence (i.e. larger SGs) favors interactions with PBs. Indeed, this interaction seems to be essential to the protection of polyadenylated mRNA during oxidative stress. Interestingly, cells depleted of G3BP1 have very similar disturbances in SG assembly and disassembly (Aulas et al., 2015). Furthermore, reintroduction of G3BP1 in TDP-43 depleted cells fully rescues SG secondary aggregation, PB docking, and mRNA protection (Aulas et al., 2012, 2015). Thus, endogenous TDP-43 regulates the primary effector of SG secondary aggregation and function, G3BP1 (Aulas et al., 2012, 2015). Intriguingly, neuronal-like cells exposed to oxidative stress demonstrated a greater vulnerability than non-neuronal cells, an effect which correlates with TDP-43 mediated regulation of G3BP1 (Aulas et al., 2012). The mechanism by which TDP-43 regulates G3BP1 remains unknown.

In contrast, depletion of FUS does not interfere with SG assembly (Aulas et al., 2012; Blechingberg et al., 2012; Sama et al., 2013). SGs form at the same rate in cells depleted of endogenous FUS compared to cells treated with control siRNA, and secondary aggregation proceeds normally (Aulas et al., 2012). Consistent with this, SG-PB docking, mRNA preservation, and cell survival is undisturbed in FUS-depleted cells following oxidative stress (Aulas et al., 2012, 2015). The role for FUS in SG disassembly is unknown. Thus, although TDP-43 and FUS are considered to be closely related, they have very divergent endogenous roles in the regulation of SG dynamics.

### Influence of TDP-43 and FUS Mutations on Stress Granule Dynamics

Whether disease-associated mutations in TDP-43 or FUS result in a gain or loss of function with respect to the role of these proteins in SG dynamics remains unresolved. Expression of ALS-linked mutations in TDP-43 is reported to increase both the number of SGs per cell and the size of individual SGs compared to cells transfected with TDP-43^WT^ (Liu-Yesucevitz et al., 2010; Dewey et al., 2011). Unfortunately, these studies do not include an analysis of untransfected cells, making it difficult to determine the impact of TDP-43 overexpression itself on SG dynamics. Notably, overexpression of TDP-43^WT^ induces cell death (Liu-Yesucevitz et al., 2010). Interestingly, mutant TDP-43 expression induces an equivalent amount of cell death in basal conditions. However, stress exposure exacerbates cell death uniquely in cells overexpressing mutant forms of TDP-43 (Liu-Yesucevitz et al., 2010). This latter result is reminiscent of that which is observed in the context of TDP-43 depletion (Aulas et al., 2012).

For FUS, the story is once again divergent. First, wild type FUS protein, both endogenous and overexpressed, localizes to SGs in response to osmotic stress but does not show a robust localization in response to oxidative, thermal or ER stress (Andersson et al., 2008; Bosco et al., 2010; Baron et al., 2013; Sama et al., 2013; Lenzi et al., 2015). However, mutant FUS expression increases the number of cells forming SGs in response to oxidative stress (Bosco et al., 2010). Arsenite-induced SGs in cells expressing the predominantly cytoplasmic mutant FUS^R495X^ are larger and modestly more abundant compared to cells expressing FUS^WT^. Moreover, SG assembly is delayed and disassembly is accelerated (Baron et al., 2013; Lenzi et al., 2015). Even though SGs are larger in FUS^R495X^ expressing cells, the dynamic exchange of TIA-1 and G3BP1 between SGs and the cytoplasm is faster compared to cells expressing FUS^WT^ (Baron et al., 2013). Thus, in the presence of mutant FUS, SG protein interactions are more labile and likely explain the observed defects in assembly and disassembly (Baron et al., 2013).

### Microtubule-based Transport in SG Dynamics: Links to ALS?

Microtubule-based transport defects are observed in several ALS models (Swarup et al., 2011; Gal et al., 2013) and thus are suggested to be relevant to ALS pathogenesis. Microtubule-directed movements are implicated in SG formation with depletion of proteins involved in microtubule-mediated transport affecting both SGs and PBs (Aizer and Shav-Tal, 2008; Aizer et al., 2008; Ohn et al., 2008; Loschi et al., 2009; Bartoli et al., 2011). In addition, microtubule destabilization leads not only to defective secondary aggregation of SGs and accelerated disassembly (Nadezhdina et al., 2010; Ivanov et al., 2011), but also increased PB formation (Bashkirov et al., 1997; Sweet et al., 2007; Aizer and Shav-Tal, 2008; Aizer et al., 2008). These same phenotypes are observed in cells depleted of TDP-43 or G3BP1. However, depletion of endogenous TDP-43 or G3BP1 is not reported to be associated with obvious alterations in the microtubule network.

Microtubule-based transport requires not only the “cytoskeletal highway” but also motor and adaptor proteins. Analysis of published data reporting on transcripts bound by TDP-43 reveals several Kinesin family members and Kinesin binding proteins (Kcl1, Kifap3, Kif3c, Kif3a, Kif5a, Kif5c, Trak1, Trak2) as well as three Dynein family members (DynII2, Dync1li2, BicD1; Polymenidou et al., 2011). While the axonal transport of mutant TDP-43 containing RNA granules is disrupted (Alami et al., 2014), the mechanism by which this occurs remains unknown. It is tempting to speculate that TDP-43 regulates the expression of these proteins, and thus influences microtubule-based transport, but this remains untested. In the context of microtubule-directed SG dynamics, TDP-43 regulates both G3BP1 and HDAC6, which together are reported to bridge the interaction between SGs and motor proteins (Kwon et al., 2007; Fiesel et al., 2010; McDonald et al., 2011). Thus, microtubule-based transport could still be relevant to the overall coordination of SG dynamics influenced by TDP-43.

The involvement of FUS in cellular movement is less studied compared to TDP-43. However, in a recent summary of FUS splicing targets, three genes reported in at least two independent studies include the actin binding protein ENAH (Protein enabled homolog), EPB4.1L2 (Band 4.1-like protein 2), and EPB4.9 (Dematin; Ishigaki et al., 2012, 2013; Lagier-Tourenne et al., 2012; Rogelj et al., 2012; Nakaya et al., 2013; Orozco and Edbauer, 2013). While the role of FUS in actin-based transport has been confirmed (Fujii and Takumi, 2005), actin-based transport is reported as not involved in SG movement (Nadezhdina et al., 2010). Interestingly, FUS has been previously observed in RNA granules transported in a microtubule-dependent manner and as a binding partner of kinesin (Kanai et al., 2004). However, whether FUS (wild type or mutant forms) can influence microtubule-based transport remains unknown.

## Unlinking Stress Granules and Pathological TDP-43 and FUS Inclusions

As described above, TDP-43 cytoplasmic inclusions are independent of inclusions marked by SG markers in AD model mice (Vanderweyde et al., 2012). However, in ALS/FTD human samples, there are a handful of examples of co-labeling of TDP-43 and FUS inclusions with well-known SG markers (Colombrita et al., 2009; Volkening et al., 2009; Dormann et al., 2010; Liu-Yesucevitz et al., 2010; Bentmann et al., 2012). Despite the many papers documenting TDP-43 positive inclusions in patient tissues, there is little known about their origins, with only a single team reporting the generation of “stable” TDP-43 positive inclusions that are distinct from SGs in cultured cells (Meyerowitz et al., 2011; Parker et al., 2012). That these inclusions are distinct from SGs is possibly in the details since the experimental conditions are quite different from those generally used to examine TDP-43 in SGs. Specifically, cells were exposed to a stress stimulus over a prolonged period of time (1 mM paraquat, 24 h) rather than the intense acute stress that is more typically used in SG studies (0.1–0.5 mM SA, 30–60 min or 42°C heat shock, 30–60 min). Interestingly, this paraquat model recapitulates the main features observed in ALS/FTD tissues, namely the nuclear clearance and cytoplasmic aggregation of TDP-43 (Meyerowitz et al., 2011; Parker et al., 2012). While the authors initially claim that the observed inclusions are SGs based on co-localization with HuR and TIAR, it is noted that two types of inclusions are actually formed by paraquat exposure. One subset is positive for TDP-43 while a second subset demonstrates robust HuR labeling. Importantly, these two types of inclusions do not share the same kinetic properties. Namely, HuR positive inclusions are cycloheximide-sensitive and disassemble once the stress is removed, consistent with a SG identity (Parker et al., 2012). However, TDP-43 positive inclusions persist in the presence of cycloheximide and after removal of paraquat, suggesting that these structures are likely not SGs according to the definition broadly accepted by the field (Kedersha et al., 1999; Parker et al., 2012). Thus, even though this stress paradigm induces TDP-43 positive inclusions, it cannot be asserted that they are truly SGs. This could be considered as *in vitro* evidence that TDP-43 pathological inclusions are distinct from SGs. Unfortunately, the functional significance of these inclusions on cell survival/vulnerability remains unknown. Similarly, the impact of FUS and its ALS-linked mutations remains to be explored. Despite these shortcomings, this paraquat model is intriguing since it mimics ALS/FTD pathology and thus may be ideally suited to further investigate whether a pathogenic link between SGs and TDP-43/FUS positive inclusions does in fact exist.

## Similarities Between TDP-43 and FUS

**FIGURE 4:**
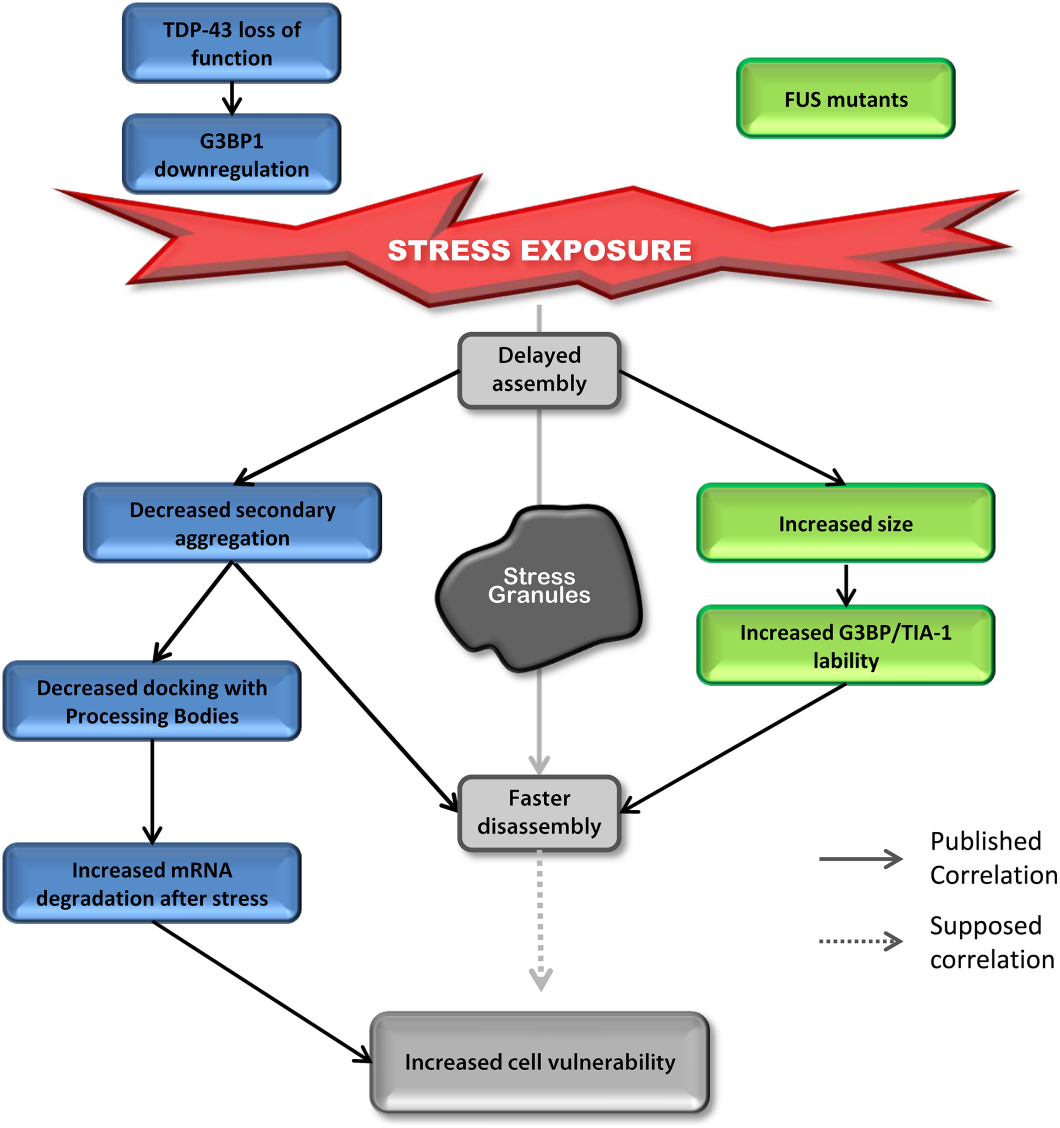
**Down-regulation of TDP-43 and expression of mutant FUS share SG phenotypes.** Delayed SG assembly and faster SG disassembly is observed in cells with reduced levels of TDP-43 as well as cells expressing mutant forms of FUS. This phenotype increases cell vulnerability. Cells reduced in TDP-43 levels will present a defect in SG secondary aggregation concomitant with a decrease in SG/PB docking followed by an increase in mRNA degradation after stress (siTDP-43 cells compared to siControl cells). This effect is mediated via G3BP1. Cells expressing mutant forms of FUS form larger SGs with more labile TIA-1 and G3BP1 protein compared to cells transfected with FUS^WT^.

Most studies on TDP-43 and FUS and SGs have investigated the “toxic gain of function” aspect of these two proteins containing ALS-linked mutations. However, surprisingly, even though the impact on assembly is well described, SG disassembly is typically ignored and the link between mutations and cell sensitivity to stress is often unexplored. In contrast, there are robust correlations between defective SG kinetics and cell vulnerability post-stress in “loss of function” models for TDP-43 that are not shared by FUS (Aulas et al., 2012, 2015). However, there are some similar alterations in SG dynamics induced by depletion of endogenous TDP-43 and overexpression of mutant FUS (**Figure 4**). Specifically, in both contexts, cells exhibit delayed SG formation and accelerated SG resolution (McDonald et al., 2011; Baron et al., 2013). Interestingly, mutant FUS expression gives rise to increased SG size which could be attributed to increased lability of TIA-1 and G3BP1 in SGs (Baron et al., 2013). Specifically, this property may result in faster disassembly due to inefficient packing/formation of individual SGs, effectively yielding larger yet less stable structures. Since there is no evidence that FUS impacts SG function and assembly, we propose that FUS mutations primarily impact SG dynamics via a gain of function mechanism. In contrast, while expression of mutant TDP-43 increases SG size, nothing is known about SG disassembly in this context, but the notion that larger SGs are more labile and thus disassemble faster, as is the case for FUS, is possible. However, TDP-43 loss of function (depletion by siRNA) induces the down-regulation of the major SG regulator G3BP1 (McDonald et al., 2011) which is linked to increased susceptibility of neuronal-like cells to oxidative stress (Aulas et al., 2012). More in-depth studies examining the impact of mutant TDP-43 on SG kinetics and G3BP1 expression/post-translational modification and/or lability in SGs are needed as is investigation of nuclear clearance of TDP-43 in the context of SGs.

The origin and nature of pathological inclusions found in ALS/FTD patients remains poorly understood. Inclusion formation could be a mechanism by which the neuron sequesters non-functional protein that could otherwise perturb normal function. Alternatively, they could arise from defective removal of normal SGs, themselves a type of cytoplasmic aggregate, responding to cellular stress. Lastly, it is remains equally possible that inefficient clearance of non-specific aggregates composed of non-functional proteins which feature aggregation-prone domains could yield these cytoplasmic accumulations or “primo-aggregates” of undefined composition. This is an interesting concept given the discovery of VCP-mediated autophagy as a mechanism to clear SGs post-heat shock (Buchan et al., 2013). However, the role of VCP remains unclear given that oxidative stress or proteasome inhibition of VCP-depleted cells feature smaller SGs with atypical composition (Seguin et al., 2014).

## Conclusion

In recent years, there has been an important increase in the novel descriptions of proteins localizing to SGs using co-localization with only one or two previously reported SG markers. However, according to the original definition of SGs, the co-localization with protein markers is not sufficient to qualify a cytoplasmic inclusion as a SG (Kedersha et al., 1999). Indeed, a large proportion of SG proteins contain a prion-like/unstructured/low complexity domain (Li et al., 2013) that could drive proteins aggregation in a non-specific way (Olszewska et al., 2012). TDP-43 and FUS most definitely have a role in the stress response via their involvement in SGs and by interacting with proteins localizing to SGs (Freibaum et al., 2010). However, determining the link between their involvement in SG dynamics/function and the formation of pathological inclusions is still very much unclear. Whether TDP-43 or FUS recruitment to pathological inclusions drives inclusion formation or they are passively incorporated via a non-specific mechanism is an important future direction. Hand in hand with this, elucidating whether these cytoplasmic inclusions are beneficial, toxic or irrelevant is of utmost importance.

## Conflict of Interest Statement

The authors declare that the research was conducted in the absence of any commercial or financial relationships that could be construed as a potential conflict of interest.
