# Supplementary material for: Alterations in stress granule dynamics driven by TDP-43 and FUS: a link to pathological inclusions in ALS?
Source: Front Cell Neurosci. 2015 Oct 23;9:423. doi: 10.3389/fncel.2015.00423 (PMC4615823; doi:10.3389/fncel.2015.00423)
Supplement: Supplementary file 1 [file Table_S1.DOCX]

**Supplemental Table 1: Proteins reported as recruited to stress granules, as assembled from 154 publications (1999-2014).**

| *Protein* | *RBP* | *Oxidative Stress* | *Thermal Stress* | *ER Stress* | *Viral Infection* | *Chemo-therapy* | *Osmotic Stress* | *Other Stress* | *PB Markers* | *Cell type* | *References* | |
| --- | --- | --- | --- | --- | --- | --- | --- | --- | --- | --- | --- | --- |
| ADAR1 | Y | sodium arsenite |  |  |  |  |  |  |  | HeLa | (Ng et al., 2013) | |
| Ago1 | Y | sodium arsenite |  |  |  |  |  |  |  | HEK293T | (Takahashi et al., 2013) | |
| Ago2 | Y |  |  |  |  | hippuristanol |  |  | Dcp1 | HeLa | (Leung et al., 2006) | |
|  |  |  |  |  |  |  |  | UV |  | HeLa | (Pothof et al., 2009) | |
|  |  | arsenic oxide |  |  |  |  |  |  |  | A343 | (Wang et al., 2010) | |
|  |  | sodium arsenite |  |  |  |  |  |  |  | HEK293T | (Takahashi et al., 2013) | |
|  |  |  |  |  |  | selenite |  |  |  | U2OS | (Fujimura et al., 2012) | |
| Angiogenin | Y | sodium arsenite |  |  |  |  |  |  |  | HeLa | (Pizzo et al., 2013) | |
| Aha1 | N |  |  |  |  | hippuristanol |  |  |  | HeLa | (Pare et al., 2009) | |
| AKAP350 | N | sodium arsenite |  |  |  |  |  |  |  | HepG2 | (Kolobova et al., 2009) | |
| Ataxin-2 | N |  | heat shock |  |  |  |  |  | (Dcp1) | DU145 | (Figley et al., 2014; Nonhoff et al., 2007) | |
|  |  | sodium arsenite |  |  |  |  |  |  | (Dcp1) | DU145 | (Figley et al., 2014; Nonhoff et al., 2007) | |
| Ataxin-2-Like | Y | sodium arsenite |  |  |  |  |  |  |  | HeLa, HEK293T | (Kaehler et al., 2014) | |
|  |  | DTT |  |  |  |  |  |  |  | HeLa | (Kaehler et al., 2014) | |
|  |  | hydrogen peroxide |  |  |  |  |  |  |  | HeLa | (Kaehler et al., 2014) | |
|  |  |  | heat shock |  |  |  |  |  |  | HeLa | (Kaehler et al., 2014) | |
|  |  |  |  |  |  |  | sorbitol |  |  | HeLa | (Kaehler et al., 2014) | |
|  |  |  |  |  |  | selenite |  |  |  | HeLa | (Kaehler et al., 2014) | |
|  |  |  |  |  |  |  |  | 5-FU |  | HeLa | (Kaehler et al., 2014) | |
| APOBEC3G | Y |  | heat shock |  |  |  |  |  |  | HeLa | (Gallois-Montbrun et al., 2007) | |
|  |  |  |  |  |  |  |  |  | Mov10, Ago1, Ago2, rck/p54, Dcp1, GW182, YB1 | HeLa | (Gallois-Montbrun et al., 2007) | |
| CCAR1 | N | sodium arsenite |  |  |  |  |  |  |  | HeLa, HepG2 | (Kolobova et al., 2009) | |
| Cdc37 | N |  |  |  |  | hippuristanol |  |  |  | HeLa | (Pare et al., 2009) | |
| CIRP | Y | sodium arsenite |  |  |  |  |  |  | (Dcp1) | NIH/3T3, COS7 | (De Leeuw et al., 2007) | |
|  |  |  |  |  |  |  | sorbitol |  |  | COS7 | (De Leeuw et al., 2007) | |
|  |  | DTT |  |  |  |  |  |  |  | COS7 | (De Leeuw et al., 2007) | |
| CNBP |  | sodium arsenite |  |  |  |  |  |  | (Dcp1) | HeLa | (Rojas et al., 2012) | |
| CUGBP-1 | Y |  | heat shock |  |  |  |  |  | (Dcp1a) | HeLa | (Fujimura et al., 2008) | |
|  |  |  |  |  |  | bortezomide |  |  |  | HeLa | (Gareau et al., 2011) | |
| CPEB1 | Y | sodium arsenite |  |  |  |  |  |  | Dcp1, rck/p54 (no stress) | HeLa | (Wilczynska et al., 2005) | |
| Caprin1 | Y | sodium arsenite |  |  |  |  |  |  |  | HeLa, NIH3T, HepG2, F11, huh7 | (Katoh et al., 2013; Kolobova et al., 2009; Kunde et al., 2011; Solomon et al., 2007) | |
|  |  |  |  |  | Japanese Encephalitis Virus 24h |  |  |  |  | huh7 | (Katoh et al., 2013) | |
| DAZL | Y |  | heat shock |  |  |  |  |  |  | testis section | (Kim et al., 2012) | |
| DDX1 | Y | sodium arsenite |  |  |  |  |  |  |  | F11, HeLa | (Kunde et al., 2011; Onishi et al., 2008) | |
| DDX3 | Y | sodium arsenite |  |  |  |  |  |  | (Dcp1) | U2OS | (Shih et al., 2012) | |
|  |  |  |  |  |  |  | sorbitol |  | (Dcp1) | U2OS | (Shih et al., 2012) | |
| DDX6 | Y |  | heat shock |  |  |  |  |  |  | DU145 | (Nonhoff et al., 2007) | |
|  |  | sodium arsenite |  |  |  |  |  |  |  | DU145 | (Nonhoff et al., 2007) | |
|  |  |  |  |  | HCV-JFH1 hepatitis C |  |  |  |  | RSc cells | (Ariumi et al., 2011) | |
|  |  |  |  |  |  |  |  | 5-FU | Dcp1 | HeLa | (Kaehler et al., 2014) | |
| DIC | N | sodium arsenite |  |  |  |  |  |  |  | COS7 | (Loschi et al., 2009) | |
| DHC | N | sodium arsenite |  |  |  |  |  |  |  | NIH3T3 | (Loschi et al., 2009) | |
| DYRK3 | N | sodium arsenite |  |  |  |  |  |  |  | HeLa | (Wippich et al., 2013) | |
|  |  |  |  |  |  |  |  | sorbitol |  | HeLa | (Wippich et al., 2013) | |
| eIF2-phospho | Y |  |  | thapsigargin |  |  |  |  |  | NIH/3T3 | (Kimball et al., 2003) | |
|  |  | sodium arsenite |  |  |  |  |  |  | (HEDLS) | Human bone marrow-derived CD34+ hematopoietic cells, HeLa | (Fujimura et al., 2009; Lavut and Raveh, 2012) | |
|  |  |  |  |  |  |  |  | 5-Fluoroudine |  | HeLa | (Kaehler et al., 2014) | |
| eIF3 | N | sodium arsenite |  |  |  |  |  |  | (Dcp1) (p54) (HEDLS) | DU145, BE-M17, NIH/3T3, HeLa, U2OS, HeLa, HEK293T, MEF, Vero cells, Human bone marrow-derived CD34+ hematopoietic cells, Sertoli cells | (Chalupnikova et al., 2008; De Leeuw et al., 2007; ErLin et al., 2015; Farny et al., 2009; Fros et al., 2012; Kedersha et al., 2002; Langereis et al., 2013; Lavut and Raveh, 2012; Liu-Yesucevitz et al., 2010; Nadezhdina et al., 2010; Ohn et al., 2008; Souquere et al., 2009) | |
|  |  |  |  |  | Semliki Forest virus infection |  |  |  |  | MEF | (McInerney et al., 2005) | |
|  |  |  |  |  | LeC virus |  |  |  |  | HEp-02 | (Hanley et al., 2010) | |
|  |  |  |  |  | TMEV, Vesicular stomatitis virus, Mammalian orthoreo-virus (MRV) |  |  |  | (Dcp1a) | HeLa | (Borghese and Michiels, 2011; Dinh et al., 2013; Qin et al., 2009) | |
|  |  |  |  | thapsigargin |  |  |  |  |  | Terato-carcinoma cells, MEF, HeLa | (Goodier et al., 2007; Langereis et al., 2013) | |
|  |  |  | heat shock |  |  |  |  |  |  | HeLa, S2, MEF | (Chalupnikova et al., 2008; Farny et al., 2009; Khong and Jan, 2011; Langereis et al., 2013; Souquere et al., 2009) | |
|  |  |  |  |  |  |  |  | clotrimazole | (Dcp1) | U2OS | (Ohn et al., 2008) | |
|  |  |  |  |  |  |  |  | pateamine A | (Dcp1) | U2OS, S2, MEF | (Farny et al., 2009; Khong and Jan, 2011; Ohn et al., 2008; Panas et al., 2012) | |
|  |  |  |  |  |  |  |  | CCCP |  | HeLa | (Qi et al., 2011) | |
|  |  |  |  |  |  |  |  | MG132 |  | MEF | (Langereis et al., 2013) | |
| eIF3a | N |  |  |  | poliovirus |  |  |  |  | HeLa | (White and Lloyd, 2011) | |
|  |  | sodium arsenite |  |  |  |  |  |  |  | huh7, HeLa | (Chudinova et al., 2012; Katoh et al., 2013) | |
| eIF3b | N | sodium arsenite |  |  |  |  |  |  | (rck) | U2OS, COS7 | (Emara et al., 2010; Hofmann et al., 2012; Li et al., 2010) | |
|  |  | hydrogen peroxide |  |  |  |  |  |  |  | U2OS | (Emara et al., 2012) | |
|  |  |  |  |  |  |  |  | clotrimazole | (rck) | COS7 | (Li et al., 2010) | |
|  |  |  | heat shock |  |  |  |  |  | (rck) | COS7, MEF | (Buchan et al., 2013; Li et al., 2010) | |
|  |  |  | cold shock |  |  |  |  |  |  | COS7 | (Hofmann et al., 2012) | |
| eIF4E | N | sodium arsenite |  |  |  |  |  |  | Dcp1 | DU145, HeLa, U2OS, Human bone marrow-derived CD34+ hematopoietic cells | (Emara et al., 2012; Emara et al., 2010; Farny et al., 2009; Fournier et al., 2013; Kedersha et al., 2002; Kedersha et al., 2005; Lavut and Raveh, 2012; Thomas et al., 2009) | |
|  |  | hydrogen peroxide |  |  |  |  |  |  |  | U2OS | (Emara et al., 2012) | |
|  |  |  |  | thapsigargin |  |  |  |  |  | NIH/3T3 | (Kimball et al., 2003) | |
|  |  |  |  |  | Mammalian ortho-reovirus (MRV), Poliovirus |  |  |  |  | HeLa | (Qin et al., 2009; White and Lloyd, 2011) | |
|  |  |  |  |  |  |  |  | pateamine A | Dcp1 | U2OS | (Dang et al., 2006; Farny et al., 2009) | |
|  |  |  |  |  |  |  |  | CCCP |  | HeLa | (Qi et al., 2011) | |
|  |  |  | heat shock |  |  |  |  |  |  | HeLa, YAMC, SR2+, MEF | (Farny et al., 2009; Hu et al., 2010; Sukarieh et al., 2009; Suzuki et al., 2009) | |
|  |  |  |  |  |  |  | sorbitol |  | (Dcp1) | U2OS | (Shih et al., 2012) | |
|  |  |  |  |  |  | bortezomide |  |  |  | HeLa | (Fournier et al., 2013; Fournier et al., 2010) | |
|  |  |  |  |  |  | selenite |  |  |  | U2OS | (Fujimura et al., 2012) | |
| eIF4A | N |  |  |  |  |  |  | pateamine A |  | U2OS, HeLa | (Dang et al., 2006; Mazroui et al., 2006) | |
|  |  |  |  |  |  |  |  | MG132 |  | HeLa | (Mazroui et al., 2007) | |
|  |  |  |  |  |  | selenite |  |  |  | U2OS | (Fujimura et al., 2012) | |
|  |  |  |  |  |  | hippuristanol |  |  |  | HeLa | (Mazroui et al., 2006) | |
|  |  | sodium arsenite |  |  |  |  |  |  |  | HeLa | (Didiot et al., 2009; Mazroui et al., 2006) | |
|  |  |  | heat shock |  |  |  |  |  |  | HeLa | (Didiot et al., 2009) | |
| eIF4B | Y |  |  |  |  |  |  | pateamine A |  | U2OS | (Dang et al., 2006) | |
|  |  |  |  |  | Poliovirus |  |  |  |  | HeLa | (White and Lloyd, 2011) | |
|  |  | sodium arsenite |  |  |  |  |  |  |  | huh7 | (Katoh et al., 2013) | |
| eIF4G | N | sodium arsenite |  |  |  |  |  |  |  | DU145, U2US, HeLa, Primary normal foreskin fibroblasts, Sertoli cells | (Arimoto et al., 2008; Brown et al., 2011; ErLin et al., 2015; Fujimura et al., 2012; Kaehler et al., 2014; Kedersha et al., 2002; Piotrowska et al., 2010) | |
|  |  |  |  | thapsigargin |  |  |  |  |  | NIH/3T3 | (Kimball et al., 2003) | |
|  |  |  |  |  | Poliovirus |  |  |  |  | HeLa | (Piotrowska et al., 2010; White et al., 2007) | |
|  |  |  | heat shock |  |  |  |  |  |  | COS7, Primary normal foreskin fibroblasts, HeLa | (Brown et al., 2011; Murata et al., 2005; Piotrowska et al., 2010; Suzuki et al., 2009) | |
|  |  |  | cold shock |  |  |  |  |  |  | COS7 | (Hofmann et al., 2012) | |
|  |  |  |  |  |  |  |  | 5-FU |  | HeLa | (Kaehler et al., 2014) | |
|  |  |  |  |  |  | selenite |  |  |  | U2OS | (Fujimura et al., 2012) | |
| eIF5a | Y | sodium arsenite |  |  |  |  |  |  | (rck) | COS7 | (Li et al., 2010) | |
|  |  |  | heat shock |  |  |  |  |  | (rck) | COS7 | (Li et al., 2010) | |
|  |  |  |  |  |  |  |  | clotrimazole | (rck) | COS7 | (Li et al., 2010) | |
|  |  |  |  |  |  | selenite |  |  |  | U2OS | (Fujimura et al., 2012) | |
| EWS | Y | sodium arsenite |  |  |  |  |  |  |  | HT-1080 | (Andersson et al., 2008) | |
|  |  |  | heat shock |  |  |  |  |  |  | HT-1080 | (Andersson et al., 2008) | |
| FAK | N |  | heat shock |  |  |  |  |  |  | P19 | (Tsai et al., 2008) | |
| FAST | N | sodium arsenite |  |  |  |  |  |  | GW182 | DU145 | (Kedersha and Anderson, 2002) | |
| FMRP | Y |  | heat shock |  |  |  |  |  |  | STEK, HeLa, P19 | (Didiot et al., 2009; Mazroui et al., 2002; Tsai et al., 2008) | |
|  |  |  |  |  |  |  |  | MG132 |  | HeLa | (Mazroui et al., 2007) | |
|  |  | sodium arsenite |  |  |  |  |  |  |  | HeLa, F11 | (Baguet et al., 2007; Didiot et al., 2009; Fournier et al., 2013; Goulet et al., 2008; Kunde et al., 2011; Linder et al., 2008) | |
|  |  |  |  |  |  |  |  | NSC 119839 |  | MEF | (Mokas et al., 2009) | |
|  |  |  |  |  |  | selenite |  |  |  | U2OS | (Fujimura et al., 2012) | |
|  |  |  |  |  |  | bortezomide |  |  |  | HeLa | (Fournier et al., 2013; Fournier et al., 2010; Gareau et al., 2011) | |
| FUS | Y | sodium arsenite |  |  |  |  |  |  | (HEDLS) | HT-1080, Primary hippo-campal neurons, HEK293T, SH-SY5Y | (Andersson et al., 2008; Bentmann et al., 2012; Blechingberg et al., 2012; Bosco et al., 2010) | |
|  |  |  |  | thapsigargin |  |  |  |  |  | HEK293T | (Bosco et al., 2010) | |
|  |  |  | heat shock |  |  |  |  |  |  | HT-1080, Primary hippo-campal neurons, SH-SY5Y, fish embryo cells, HEK293T | (Andersson et al., 2008; Bentmann et al., 2012; Bosco et al., 2010) | |
|  |  |  |  |  |  |  |  | clotrimazole |  | SH-SY5Y, Primary hippo-campal neurons | (Bentmann et al., 2012) | |
|  |  |  |  |  |  |  | sorbitol |  |  | HeLa | (Sama et al., 2013) | |
| FXR1 | Y |  | heat shock |  |  |  |  |  |  | STEK, HeLa, Ha-Cat | (Hofmann et al., 2006; Mazroui et al., 2002) | |
|  |  | sodium arsenite |  |  |  |  |  |  |  | HeLa, NIH3T3, Ha-Cat, IDH4 | (Fournier et al., 2013; Hofmann et al., 2006; Lian and Gallouzi, 2009; Sahoo et al., 2012) | |
|  |  |  |  |  |  |  |  | pateamine A |  | U2OS | (Dang et al., 2006) | |
|  |  |  |  |  |  |  |  | MG132 |  | HeLa, MEF | (Mazroui et al., 2007) | |
|  |  |  |  |  |  |  |  | NSC 119839 |  | MEF | (Mokas et al., 2009) | |
|  |  |  |  |  |  | bortezomide |  |  |  | HeLa | (Fournier et al., 2013; Fournier et al., 2010) | |
| FXR2P | Y |  | heat shock |  |  |  |  |  |  | STEK, HeLa | (Mazroui et al., 2002) | |
| G3BP1 | Y |  | heat shock |  |  |  |  |  |  | HeLa, Ha-Cat, MEF, NMuMG | (Bann et al., 2014; Deigendesch et al., 2006; Gao et al., 2010; Hofmann et al., 2006; Hua and Zhou, 2004; Kwon et al., 2007; Langereis et al., 2013; Sama et al., 2013; Sukarieh et al., 2009) | |
|  |  |  | cold shock |  |  |  |  |  |  | COS7 | (Hofmann et al., 2012) | |
|  |  | sodium arsenite |  |  |  |  |  |  |  | HeLa, 3T3, Ha-Cat, RAS, HepG2, IDH4, U2OS, HEK293T, Jurkat, HuH7, Vero cells | (Deigendesch et al., 2006; Emara et al., 2010; Figley et al., 2014; Fournier et al., 2013; Fros et al., 2012; Fujimura et al., 2009; Fujimura et al., 2012; Gao et al., 2010; Goulet et al., 2008; Hinton et al., 2010; Hofmann et al., 2006; Katoh et al., 2013; Kolobova et al., 2009; Kwon et al., 2007; Lian and Gallouzi, 2009; Matsuki et al., 2013; Mazroui et al., 2006; Onomoto et al., 2012; Qi et al., 2011; Sama et al., 2013; Seguin et al., 2014; Singleton et al., 2014; Solomon et al., 2007; Takahashi et al., 2013; Tourriere et al., 2003; Wehner et al., 2010; Weissbach and Scadden, 2012; Wen et al., 2014) | |
|  |  | arsenic acid |  |  |  |  |  |  |  | HeLa | (Langereis et al., 2013) | |
|  |  | Hydrogen peroxide |  |  |  |  |  |  |  | HeLa, U2OS | (Emara et al., 2012; Sama et al., 2013) | |
|  |  |  |  |  |  | hippuristanol |  |  |  | HeLa, MEF | (Mazroui et al., 2006) | |
|  |  |  |  |  |  | bortezomide |  |  |  | HeLa | (Fournier et al., 2013; Fournier et al., 2010; Gareau et al., 2011) | |
|  |  |  |  |  |  | selenite |  |  |  | U2OS | (Fujimura et al., 2012) | |
|  |  |  |  |  | Poliovirus |  |  |  |  | HeLa, HEK293T, MCF7, Vero cells | (White et al., 2007) | |
|  |  |  |  |  | RVS virus |  |  |  |  | HEp-2 | (Lindquist et al., 2010) | |
|  |  |  |  |  | HCV-JFH1 hepatitis C |  |  |  |  | RSc cells, HuH7 | (Ariumi et al., 2011; Garaigorta et al., 2012) | |
|  |  |  |  |  | Mammalian orthoreo-virus (MRV), Coxsackie virus B3 (CVB3), Mengovirus (Zn domain mutant) |  |  |  |  | HeLa | (Fung et al., 2013; Langereis et al., 2013; Qin et al., 2009) | |
|  |  |  |  |  | Mengovirus |  |  |  |  | MEF | (Langereis et al., 2013) | |
|  |  |  |  |  | Measles virus |  |  |  |  | HeLa | (Okonski and Samuel, 2013) | |
|  |  |  |  |  | Japanese Encephalitis Virus 24h |  |  |  |  | huh7 | (Katoh et al., 2013) | |
|  |  |  |  |  | Semliki Forest virus infection 8h |  |  |  |  | MEF | (Panas et al., 2012) | |
|  |  |  |  |  | Chikungunya |  |  |  |  | Vero cells | (Fros et al., 2012) | |
|  |  |  |  |  | West Nile Virus |  |  |  |  | BHK | (Courtney et al., 2012) | |
|  |  |  |  | thapsigargin |  |  |  |  |  | HeLa | (Sama et al., 2013; Wehner et al., 2010) | |
|  |  |  |  |  |  |  |  | NSC 119839 |  | MEF | (Mokas et al., 2009) | |
|  |  |  |  |  |  |  |  | carbonyl cyanide 3-chlorophenylhydrazone |  | HeLa | (Qi et al., 2011) | |
|  |  |  |  |  |  |  |  | MG132 |  | HeLa | (Mazroui et al., 2007) | |
|  |  |  |  |  |  |  |  | CCCP |  | HeLa | (Kwon et al., 2007) | |
|  |  |  |  |  |  |  |  | UV |  | HeLa | (Kwon et al., 2007) | |
|  |  |  |  |  |  |  |  | pateamine A |  | HeLa | (Mazroui et al., 2006) | |
|  |  |  |  |  |  |  |  | 5-FU |  | HeLa | (Kaehler et al., 2014) | |
|  |  |  |  |  |  |  | sorbitol |  |  | HeLa | (Sama et al., 2013) | |
| G3BP2 | Y | sodium arsenite |  |  |  |  |  |  |  | HeLa | (Matsuki et al., 2013) | |
|  |  |  | heat shock |  |  |  |  |  | (Dcp1) | SK-N-BE | (Kobayashi et al., 2012) | |
| Ge-1 | Y |  |  |  |  |  |  |  |  |  | (Yu et al., 2005) | |
| Grb7 | Y |  | heat shock |  |  |  |  |  |  | P19 | (Tsai et al., 2008) | |
| HDAC6 | N |  |  |  |  |  |  | CCCP |  | HeLa | (Kwon et al., 2007) | |
|  |  | sodium arsenite |  |  |  |  |  |  |  | HeLa | (Kwon et al., 2007) | |
|  |  |  |  |  |  |  |  | UV |  | HeLa | (Kwon et al., 2007) | |
|  |  |  | heat shock |  |  |  |  |  |  | HeLa | (Kwon et al., 2007) | |
| hnRNPA1 | Y |  | heat shock |  |  |  |  |  |  | NIH 3T3 | (Guil et al., 2006) | |
|  |  | sodium arsenite |  |  |  |  |  |  |  | NIH 3T3 | (Guil et al., 2006; Lee et al., 2014) | |
|  |  |  |  |  |  |  | sorbitol |  |  | NIH 3T3, cortical glia, HEK293T, terato-carcinoma cells | (Dewey et al., 2011; Goodier et al., 2007; Guil et al., 2006) | |
| hnRNPA2 | Y | sodium arsenite |  |  |  |  |  |  |  | HeLa | (McDonald et al., 2011) | |
| hnRNPK | Y |  |  |  |  |  | sorbitol |  |  | MTD-1 | (Fukuda et al., 2009) | |
|  |  | sodium arsenite |  |  |  |  |  |  | (Dcp1) | MTD-1 | (Fukuda et al., 2009) | |
| hnRNPQ | Y |  |  | thapsigargin |  |  |  |  | GW182 | HeLa | (Quaresma et al., 2009) | |
|  |  |  | heat shock |  |  |  |  |  | GW182 | COS7 | (Quaresma et al., 2009) | |
|  |  | sodium arsenite |  |  |  |  |  |  | GW182 | COS7, HuH7 | (Katoh et al., 2013; Quaresma et al., 2009) | |
|  |  |  |  |  | Japanese Encephalitis Virus 24h |  |  |  |  | Huh7 | (Katoh et al., 2013) | |
| HOP | N |  |  |  |  | hippuristanol |  |  |  | HeLa | (Pare et al., 2009) | |
| HSP27 | N |  | heat shock |  |  |  |  |  |  | DU145, HeLa | (Kedersha et al., 1999; Piotrowska et al., 2010) | |
| HSP90 | N |  |  |  |  | hippuristanol |  |  |  | HeLa | (Pare et al., 2009) | |
| HuD | Y |  | heat shock |  |  |  |  |  |  | PC12 | (Burry and Smith, 2006) | |
| HuR | Y | sodium arsenite |  |  |  |  |  |  |  | DU145, NSC34, RAS, HeLa, IDH4, NRK, NIH3T, oligoden-drocytes, Sertoli cells | (Bounedjah et al., 2014; Chalupnikova et al., 2008; Chang and Tarn, 2009; Colombrita et al., 2009; ErLin et al., 2015; Figley et al., 2014; Kedersha et al., 2002; Lian and Gallouzi, 2009; Mahboubi et al., 2013; Sahoo et al., 2012; Thomas et al., 2005) | |
|  |  | diethyl maleate |  |  |  |  |  |  |  | HeLa | (Mahboubi et al., 2013) | |
|  |  | paraquat |  |  |  |  |  |  |  | HeLa | (Parker et al., 2012) | |
|  |  |  |  |  |  |  | sorbitol |  |  | HEK293T | (Dewey et al., 2011) | |
|  |  |  |  |  |  |  | sorbitol |  |  | cortical glia | (Dewey et al., 2011) | |
|  |  |  | heat shock |  |  |  |  |  |  | NSC34, NMuMG, COS7, P19, HeLa | (Chalupnikova et al., 2008; Colombrita et al., 2009; Gallois-Montbrun et al., 2007; Mahboubi et al., 2013; Murata et al., 2005; Tsai et al., 2008) | |
|  |  |  |  |  |  |  |  | MG132 |  | NSC34, HeLa, MEF | (Colombrita et al., 2009; Mazroui et al., 2007) | |
|  |  |  |  |  |  |  |  | FCCP |  | DU145, HeLa, COS7 | (Stoecklin et al., 2004) | |
|  |  |  |  |  |  |  |  | pateamine A |  | HeLa, MEF | (Fujimura et al., 2012; Mazroui et al., 2006) | |
|  |  |  |  |  |  |  |  | NSC 119839 |  | MEF | (Mokas et al., 2009) | |
|  |  |  |  |  |  | selenite |  |  |  | U2OS | (Fujimura et al., 2012) | |
|  |  |  |  |  |  | hippuristanol |  |  |  | HeLa, MEF | (Mazroui et al., 2006) | |
|  |  |  |  |  |  | bortezomide |  |  |  | HeLa | (Fournier et al., 2010) | |
|  |  |  |  |  | RVS virus |  |  |  |  | HEp-2 | (Lindquist et al., 2010) | |
|  |  |  |  |  | CVB3 |  |  |  |  | HeLa | (Fung et al., 2013) | |
| IGF2BP3 | Y |  | heat shock |  |  |  |  |  |  | SK-N-BE | (Kobayashi et al., 2012) | |
| importin alpha1 | N | sodium arsenite |  |  |  |  |  |  |  | HeLa | (Fujimura et al., 2010; Mahboubi et al., 2013) | |
|  |  |  | heat shock |  |  |  |  |  |  | HeLa | (Fujimura et al., 2010; Mahboubi et al., 2013) | |
|  |  |  |  |  |  | hippuristanol |  |  |  | HeLa | (Fujimura et al., 2010) | |
|  |  | diethyl maleate |  |  |  |  |  |  | (Dcp1) | HeLa | (Mahboubi et al., 2013) | |
| importin alpha4 | N | diethyl maleate |  |  |  |  |  |  | (Dcp1) | HeLa | (Mahboubi et al., 2013) | |
|  |  | sodium arsenite |  |  |  |  |  |  |  | HeLa | (Mahboubi et al., 2013) | |
|  |  |  | heat shock |  |  |  |  |  |  | HeLa | (Mahboubi et al., 2013) | |
| importin alpha 5 | N | diethyl maleate |  |  |  |  |  |  | (Dcp1) | HeLa | (Mahboubi et al., 2013) | |
|  |  | sodium arsenite |  |  |  |  |  |  |  | HeLa | (Mahboubi et al., 2013) | |
|  |  |  | heat shock |  |  |  |  |  |  | HeLa | (Mahboubi et al., 2013) | |
| importin beta1 | N | diethyl maleate |  |  |  |  |  |  |  | HeLa | (Mahboubi et al., 2013) | |
|  |  | sodium arsenite |  |  |  |  |  |  | (Dcp1a) | HeLa | (Chang and Tarn, 2009; Mahboubi et al., 2013) | |
| phopho-map | N | sodium arsenite |  |  |  |  |  |  | Dcp1 | HEK293T | (Wasserman et al., 2010) | |
|  |  |  | heat shock |  |  |  |  |  | Dcp1 | HEK293T | (Wasserman et al., 2010) | |
| KHC | N | sodium arsenite |  |  |  |  |  |  |  | NIH3T3 | (Loschi et al., 2009) | |
| KLC | N | sodium arsenite |  |  |  |  |  |  |  | NIH3T3 | (Loschi et al., 2009) | |
| LC3 | N |  |  |  |  |  |  | MG132 |  | HeLa | (Seguin et al., 2014) | |
|  |  | sodium arsenite |  |  |  |  |  |  |  | HeLa | (Seguin et al., 2014) | |
|  |  |  | heat shock |  |  |  |  |  |  | HeLa | (Seguin et al., 2014) | |
| Lin28 | Y |  | heat shock |  |  |  |  |  | Dcp1 | P19 | (Balzer and Moss, 2007) | |
| LGP2 |  | sodium arsenite |  |  |  |  |  |  |  | HeLa | (Onomoto et al., 2012) | |
| MBNL1 | Y | sodium arsenite |  |  |  |  |  |  | (Dcp1a) | HeLa, COS7 | (Onishi et al., 2008) | |
| MLN51 | Y | sodium arsenite |  |  |  |  |  |  | (Dcp1) | HeLa | (Baguet et al., 2007; Didiot et al., 2009) | |
| Musashi1 |  |  | heat shock |  |  |  |  |  |  | Sertoli cells | (ErLin et al., 2015) | |
| NF90 | Y | sodium arsenite |  |  |  |  |  |  |  | HEK293 | (Wen et al., 2014) | |
| OGG1 | N |  |  |  |  |  |  | cadmium chloride |  | MCF7 | (Bravard et al., 2010) | |
| OAS | Y | sodium arsenite |  |  |  |  |  |  |  | HeLa | (Onomoto et al., 2012) | |
| OGFOD1 | N | sodium arsenite |  |  |  |  |  |  |  | HeLa | (Wehner et al., 2010) | |
|  |  |  |  | thapsigargin |  |  |  |  |  | HeLa | (Wehner et al., 2010) | |
| OGN | N |  |  |  |  |  |  | clotrimazole | (Dcp1) | U2OS | (Ohn et al., 2008) | |
|  |  |  |  |  |  |  |  | pateamine A | (Dcp1) | U2OS | (Ohn et al., 2008) | |
|  |  | sodium arsenite |  |  |  |  |  |  | (Dcp1) | U2OS | (Ohn et al., 2008) | |
| PABP | Y |  | heat shock |  |  |  |  |  |  | STEK, HeLa, SR2+, S2, COS7, NMuMG, SK-N-BE | (Bann et al., 2014; Farny et al., 2009; Gallois-Montbrun et al., 2007; Hofmann et al., 2012; Kobayashi et al., 2012; Mazroui et al., 2002) | |
|  |  |  |  |  |  |  |  | pateamine A |  | U2OS, HeLa, S2 | (Dang et al., 2006; Khong and Jan, 2011; Mazroui et al., 2006) | |
|  |  |  |  |  |  |  |  | MG132 |  | HeLa | (Mazroui et al., 2007) | |
|  |  |  |  |  |  |  |  | CCCP |  | HeLa | (Qi et al., 2011) | |
|  |  |  |  |  |  | hippuristanol |  |  |  | HeLa | (Mazroui et al., 2006) | |
|  |  |  |  |  |  | selenite |  |  |  | U2OS | (Fujimura et al., 2012) | |
|  |  |  |  | thapsigargin |  |  |  |  |  | Terato-carcinoma cells | (Goodier et al., 2007) | |
|  |  |  |  |  | Poliovirus |  |  |  |  | HeLa | (White et al., 2007) | |
|  |  | sodium arsenite |  |  |  |  |  |  |  | HEK293T, oligoden-drocytes, HeLa, SR2+, COS7, S2, U2OS, Jurkat, SK-N-BE | (Chang and Tarn, 2009; Emara et al., 2012; Farny et al., 2009; Henao-Mejia et al., 2009; Khong and Jan, 2011; Kobayashi et al., 2012; Loschi et al., 2009; Matsuki et al., 2013; Nadezhdina et al., 2010; Takahashi et al., 2013; Thomas et al., 2005; Wen et al., 2014; Wippich et al., 2013) | |
|  |  | hydrogen peroxide |  |  |  |  |  |  |  | U2OS | (Emara et al., 2012) | |
|  |  |  |  |  |  |  | sortbitol |  | (Dcp1) | HeLa, U2OS | (Shih et al., 2012; Wippich et al., 2013) | |
| PACT | Y |  |  |  |  | hippuristanol |  |  |  | HeLa | (Pare et al., 2009) | |
| PCBP2 | Y | sodium arsenite |  |  |  |  |  |  | (Dcp1) | HeLa | (Fujimura et al., 2008) | |
|  |  |  | heat shock |  |  |  |  |  |  | HeLa | (Fujimura et al., 2008) | |
|  |  | DTT |  |  |  |  |  |  |  | HeLa | (Fujimura et al., 2008) | |
| PKC | N |  | heat shock |  |  |  |  |  |  | SK-N-BE | (Kobayashi et al., 2012) | |
|  |  | sodium arsenite |  |  |  |  |  |  |  | SK-N-BE | (Kobayashi et al., 2012) | |
| PKR | Y | sodium arsenite |  |  |  |  |  |  |  | HeLa | (Onomoto et al., 2012) | |
| PKP3 | N |  | heat shock |  |  |  |  |  |  | Ha-Cat | (Hofmann et al., 2006) | |
|  |  | sodium arsenite |  |  |  |  |  |  |  | Ha-Cat | (Hofmann et al., 2006) | |
| PMRT |  | sodium arsenite |  |  |  |  |  |  |  | SH-SY5Y | (Yamaguchi and Kitajo, 2012) | |
| PQBP1 | N | sodium arsenite |  |  |  |  |  |  |  | Primary neurons | (Kunde et al., 2011) | |
| profilin-1 | N | sodium arsenite |  |  |  |  |  |  |  | HeLa | (Figley et al., 2014) | |
|  |  |  | heat shock |  |  |  |  |  |  | HeLa | (Figley et al., 2014) | |
| profilin-2 | N | sodium arsenite |  |  |  |  |  |  |  | HeLa | (Figley et al., 2014) | |
| QKI-5 | Y | arsenic oxide |  |  |  |  |  |  |  | A343 | (Wang et al., 2010) | |
| QKI-6 | Y | arsenic oxide |  |  |  |  |  |  |  | A343, primary rat oligoden-drocytes | (Wang et al., 2010) | |
| Ran | Y | sodium arsenite |  |  |  |  |  |  |  | HeLa | (Fujimura et al., 2010) | |
|  |  |  | heat shock |  |  |  |  |  |  | HeLa | (Fujimura et al., 2010) | |
|  |  |  |  |  |  | hippuristanol |  |  |  | HeLa | (Fujimura et al., 2010) | |
| RAP55 | Y | sodium arsenite |  |  |  |  |  |  | Dcp1 | Hep-2 | (Mok et al., 2012) | |
|  |  |  |  |  | Influenza virus NS1 |  |  |  |  | HEK293T | (Mok et al., 2012) | |
| RACK1 | Y | sodium arsenite |  |  |  |  |  |  | Dcp1 | HeLa, HEK293T | (Arimoto et al., 2008; Takahashi et al., 2013) | |
|  |  |  |  |  |  |  |  | 5-FU |  | HeLa | (Kaehler et al., 2014) | |
| RBM4 | Y | sodium arsenite |  |  |  |  |  |  |  | HeLa | (Lin et al., 2007) | |
| RBM42 | Y |  |  |  |  |  | sorbitol |  |  | MTD-1 | (Fukuda et al., 2009) | |
|  |  | sodium arsenite |  |  |  |  |  |  | (Dcp1) | MTD-1 | (Fukuda et al., 2009) | |
| RHAU | Y |  |  |  |  |  |  | CCCP |  | HeLa | (Chalupnikova et al., 2008) | |
|  |  |  |  |  |  | hippuristanol |  |  |  | HeLa | (Chalupnikova et al., 2008) | |
|  |  | sodium arsenite |  |  |  |  |  |  |  | HeLa | (Chalupnikova et al., 2008) | |
|  |  |  | heat shock |  |  |  |  |  |  | HeLa | (Chalupnikova et al., 2008) | |
| RIG-1 | Y | sodium arsenite |  |  |  |  |  |  |  | HeLa | (Onomoto et al., 2012) | |
| RhoA | N |  | heat shock |  |  |  |  |  |  | P19 | (Tsai et al., 2008) | |
| RNase-L | Y | sodium arsenite |  |  |  |  |  |  |  | HeLa | (Onomoto et al., 2012) | |
| RNH1 | Y | sodium arsenite |  |  |  |  |  |  |  | HeLa | (Pizzo et al., 2013) | |
| ROCK1 | N |  | heat shock |  |  |  |  |  |  | P19 | (Tsai et al., 2008) | |
| roquin | Y | sodium arsenite |  |  |  |  |  |  |  | HEK293T, Jurkat | (Athanasopoulos et al., 2010) | |
| RSK2 | N | sodium arsenite |  |  |  |  |  |  |  | MCF7, MDA-MB-231, T47D, MCF-10A, HME | (Eisinger-Mathason et al., 2008) | |
| ribosomal subunit S3 | N |  |  | thapsigargin |  |  |  |  |  | NIH/3T3 | (Kimball et al., 2003) | |
| ribosomal subunit S6 | N |  |  | thapsigargin |  |  |  |  |  | NIH/3T3 | (Kimball et al., 2003) | |
|  |  |  |  |  |  |  |  | pateamine A |  | U2OS | (Dang et al., 2006) | |
|  |  | sodium arsenite |  |  |  |  |  |  |  | Oligoden-drocytes | (Thomas et al., 2009) | |
| ribosomal subunit S18 | N | sodium arsenite |  |  |  |  |  |  |  | SR2+ | (Farny et al., 2009) | |
|  |  |  | heat shock |  |  |  |  |  |  | SR2+ | (Farny et al., 2009) | |
| ribosomal subunit S19 | N | sodium arsenite |  |  |  |  |  |  |  | DU145 | (Kedersha et al., 2002) | |
| Sam68 | Y | sodium arsenite |  |  |  |  |  |  |  | HEK293T, HeLa | (Henao-Mejia et al., 2009; Piotrowska et al., 2010) | |
|  |  |  |  |  | Poliovirus 4h |  |  |  |  | HeLa | (Piotrowska et al., 2010) | |
|  |  |  | heat shock |  |  |  |  |  |  | HeLa | (Piotrowska et al., 2010) | |
| SERBP1 | Y | sodium arsenite |  |  |  |  |  |  |  | HeLa | (Lee et al., 2014) | |
| Smg1 | Y |  |  |  |  |  |  |  |  | COS7, HeLa, BHK | (Baez and Boccaccio, 2005) | |
|  |  | hydrogen peroxide |  |  |  |  |  |  | (Dcp1) | Primary normal foreskin fibroblasts, | (Brown et al., 2011) | |
|  |  |  | heat shock |  |  |  |  |  |  | Primary normal foreskin fibroblasts | (Brown et al., 2011) | |
| SMN | Y |  | heat shock |  |  |  |  |  |  | HeLa | (Hua and Zhou, 2004) | |
|  |  | sodium arsenite |  |  |  |  |  |  |  | HeLa | (Hua and Zhou, 2004) | |
|  |  |  |  |  |  |  |  | cadmium chloride |  | MCF7 | (Bravard et al., 2010) | |
| SGNP | Y | sodium arsenite |  |  |  |  |  |  |  | LHCNM2 human myoblasts | (Zhu et al., 2008) | |
| SRp20 |  |  |  |  | Poliovirus infection 3h |  |  |  |  | SK-N-SH | (Fitzgerald and Semler, 2013) | |
| Stau1 | Y |  |  | thapsigargin |  |  |  |  | (Dcp1) | NIH/3T3 | (Thomas et al., 2009) | |
|  |  | sodium arsenite |  |  |  |  |  |  |  | HeLa, Oligoden-drocytes, NIH3T | (Loschi et al., 2009; Thomas et al., 2009) | |
|  |  |  |  |  |  | hippuristanol |  |  |  | U2OS | (Thomas et al., 2009) | |
| stau2 | Y | sodium arsenite |  |  |  |  |  |  |  | Oligoden-drocytes | (Thomas et al., 2009) | |
| T1L | N |  |  |  | Mammalian ortho-reovirus (MRV) infection 2-6h |  |  |  | (Dcp1) | HeLa | (Qin et al., 2009) | |
| TAF15 | Y | sodium arsenite |  |  |  |  |  |  |  | HT-1080, HEK293T | (Andersson et al., 2008; Blechingberg et al., 2012) | |
|  |  |  | heat shock |  |  |  |  |  |  | HT-1080 | (Andersson et al., 2008) | |
| TDP-43 | Y |  |  |  |  |  | sorbitol |  |  | HEK293T, cortical glia | (Dewey et al., 2011) | |
|  |  |  |  |  |  |  | Hanks balanced salt solution |  |  | HEK293, BE-M17 | (Liu-Yesucevitz et al., 2010) | |
|  |  | sodium arsenite |  |  |  |  |  |  | (GW182) | BE-M17, HEK293T, NSC34, HeLa, SK-N-SH | (Colombrita et al., 2009; Liu-Yesucevitz et al., 2010; McDonald et al., 2011) | |
|  |  |  | heat shock |  |  |  |  |  |  | HeLa, NSC34 | (Colombrita et al., 2009; McDonald et al., 2011) | |
|  |  |  |  |  |  |  |  | MG132 |  | NSC34 | (Colombrita et al., 2009) | |
|  |  |  |  | thapsigargin |  |  |  |  |  | HeLa | (McDonald et al., 2011) | |
| Tdrd3 | N | sodium arsenite |  |  |  |  |  |  | (GW182) | HeLa | (Emara et al., 2010; Linder et al., 2008) | |
|  |  |  | heat shock |  |  |  |  |  |  | HeLa | (Emara et al., 2010) | |
|  |  |  |  |  |  |  | sorbitol |  |  | HeLa | (Goulet et al., 2008) | |
| TIA-1 | Y |  | heat shock |  |  |  |  |  | Dcp1 | DU145, HeLa, Bladder carcinoma cell, normal uro-epithelial cells, NIH3T3, bladder cancer cell lines 5637 and 639v, A549, testis section, COS7, NSC34, P19, HEK293T, SH-SY5Y, YAMC, Primary hippo-campal neurons | (Balzer and Moss, 2007; Bentmann et al., 2012; Carpio et al., 2013; Carpio et al., 2010; Chalupnikova et al., 2008; Colombrita et al., 2009; Didiot et al., 2009; Fujimura et al., 2008; Fujimura et al., 2010; Gallois-Montbrun et al., 2007; Gao et al., 2010; Goulet et al., 2008; Hu et al., 2010; Kedersha et al., 1999; Kim et al., 2012; Lindsay and McCaffrey, 2011; McDonald et al., 2011; Murata et al., 2005; Nikpour et al., 2011; Nonhoff et al., 2007; Piotrowska et al., 2010; Quaresma et al., 2009; Seguin et al., 2014; Tsai et al., 2008; Wasserman et al., 2010; Zhang et al., 2014) | |
|  |  | sodium arsenite |  |  |  |  |  |  | (GW182, HEDLS), Dcp1 | COS7, BE-M17, U2OS, NSC34, DU145, HeLa, NIH3T3, MDA-MB-231, MCF-10A, T47D, HME, HEK293T, Primary hippo-campal neurons, SH-SY5Y, Jurkat, RES, Vero, F11, HuH7, SK-N-SH, NG108-15, Human bone marrow-derived CD34+ hemato-poietic cells | (Athanasopoulos et al., 2010; Aulas et al., 2012; Bentmann et al., 2012; Blechingberg et al., 2012; Brown et al., 2011; Carpio et al., 2010; Chalupnikova et al., 2008; Chang and Tarn, 2009; Colombrita et al., 2009; Didiot et al., 2009; Eisinger-Mathason et al., 2008; Emara et al., 2012; Fitzgerald and Semler, 2013; Fujimura et al., 2009; Fujimura et al., 2010; Goulet et al., 2008; Katoh et al., 2013; Kedersha et al., 2002; Kedersha et al., 1999; Kunde et al., 2011; Lavut and Raveh, 2012; Lee et al., 2014; Linero et al., 2011; Liu-Yesucevitz et al., 2010; McDonald et al., 2011; Nadezhdina et al., 2010; Nonhoff et al., 2007; Ohn et al., 2008; Onishi et al., 2008; Piotrowska et al., 2010; Quaresma et al., 2009; Seguin et al., 2014; Solomon et al., 2007; Souquere et al., 2009; Tanaka et al., 2014; Unsworth et al., 2010; Wasserman et al., 2010; Zhang et al., 2011) | |
|  |  | hydrogen peroxide |  |  |  |  |  |  |  | Primary normal foreskin fibroblasts | (Brown et al., 2011) | |
|  |  | hydrogen peroxide |  |  |  |  |  |  |  | U2OS | (Emara et al., 2012) | |
|  |  | arsenic oxide |  |  |  |  |  |  |  | primary rat oligoden-drocytes | (Wang et al., 2010) | |
|  |  |  |  | thapsigargin |  |  |  |  |  | HeLa, NIH3T3, ma cells | (Goodier et al., 2007; Kimball et al., 2003; McDonald et al., 2011; Quaresma et al., 2009) | |
|  |  |  |  |  |  |  |  | FCCP |  | DU145 | (Kedersha et al., 2002) | |
|  |  |  |  |  |  |  |  | DPI |  | DU145 | (Kedersha et al., 2002) | |
|  |  |  |  |  |  |  |  | oligomycin |  | DU145 | (Balzer and Moss, 2007) | |
|  |  |  |  |  |  |  |  | FCCP |  | DU145, HeLa, COS7 | (Stoecklin et al., 2004) | |
|  |  |  |  |  |  |  |  | pateamine A | Dcp1 | U2OS, HeLa, MEF | (Dang et al., 2006; Mazroui et al., 2006; Panas et al., 2012) | |
|  |  |  |  |  |  |  |  | guanabenz |  | HuH7 | (Ruggieri et al., 2012) | |
|  |  |  |  |  |  |  |  | MG132 |  | NSC34, HeLa | (Colombrita et al., 2009; Mazroui et al., 2007) | |
|  |  |  |  |  |  |  |  | CCCP |  | HeLa | (Chalupnikova et al., 2008) | |
|  |  |  |  |  |  |  |  | clotrimazole |  | Primary hippo-campal neurons, SH-SY5Y | (Bentmann et al., 2012) | |
|  |  |  |  |  |  |  |  | MG132 |  | HeLa | (Schwartz et al., 2012) | |
|  |  |  |  |  |  |  |  | UV |  | HeLa, NIH3T3 | (Carpio et al., 2010; Pothof et al., 2009) | |
|  |  |  |  |  |  |  | sorbitol |  |  | HeLa | (Goulet et al., 2008) | |
|  |  |  |  |  |  | hippuristanol |  |  | Dcp1 | HeLa, U2OS, A549 | (Chalupnikova et al., 2008; Fujimura et al., 2010; Leung et al., 2006; Mazroui et al., 2006; Pare et al., 2009; Thomas et al., 2009; Zhang et al., 2014) | |
|  |  |  |  |  | Semliki Forest virus infection |  |  |  |  | MEF | (McInerney et al., 2005) | |
|  |  |  |  |  | West Nile virus |  |  |  |  | BHK | (Emara and Brinton, 2007) | |
|  |  |  |  |  | Mammalian ortho-reovirus (MRV) infection 2-6h |  |  |  |  | MEF, HeLa | (Qin et al., 2009) |  |
|  |  |  |  |  | Poliovirus |  |  |  |  | COS7, HeLa | (Piotrowska et al., 2010) | |
|  |  |  |  |  | RVS virus |  |  |  |  | HEp-2 | (Lindquist et al., 2010) | |
|  |  |  |  |  | TMEV |  |  |  |  | HeLa | (Borghese and Michiels, 2011) | |
|  |  |  |  |  | Poliovirus |  |  |  |  | SK-N-SH, HeLa | (Fitzgerald and Semler, 2013; White and Lloyd, 2011) | |
|  |  |  |  |  | CVB3 |  |  |  |  | HeLa | (Fung et al., 2013) | |
|  |  |  |  |  | Mengovirus |  |  |  |  | HeLa | (Langereis et al., 2013) | |
|  |  |  |  |  | Japanese Encephalitis Virus 24h |  |  |  |  | huh7 | (Katoh et al., 2013) | |
|  |  |  |  |  | Semliki Forest virus infection 8h |  |  |  |  | MEF | (Panas et al., 2012) | |
|  |  |  |  |  | Vesicular stomatitis virus 5h |  |  |  |  | HeLa | (Dinh et al., 2013) | |
|  |  |  |  |  | Hepatitis C |  |  |  |  | HuH7 | (Garaigorta et al., 2012) | |
|  |  |  |  |  | Herpes simplex virus 2 8-20h |  |  |  |  | HeLa | (Finnen et al., 2012) | |
| TIAR | Y |  | heat shock |  |  |  |  |  |  | DU145, HeLa, U2OS, COS7, HEK293T, MEF | (Bosco et al., 2010; Buchan et al., 2013; Hua and Zhou, 2004; Kedersha et al., 1999; Murata et al., 2005; Stohr et al., 2006) | |
|  |  | sodium arsenite |  |  |  |  |  |  | (Dcp1, Dcp2) | COS7, BE-M17, HeLa, U2OS, MTD-1, HEK293T, NIH3T3, L929, SH-SY5Y, LHCNM2 human myoblasts, oligoden-drocytes, Sertoli cells | (Bosco et al., 2010; De Leeuw et al., 2007; ErLin et al., 2015; Fujimura et al., 2012; Fukuda et al., 2009; Hua and Zhou, 2004; Kedersha et al., 1999; Linder et al., 2008; Liu-Yesucevitz et al., 2010; Loschi et al., 2009; Ng et al., 2013; Qin et al., 2009; Rojas et al., 2012; Stohr et al., 2006; Thomas et al., 2005; Weissbach and Scadden, 2012; Yamaguchi and Kitajo, 2012; Zhu et al., 2008) | |
|  |  |  |  |  | West Nile virus |  |  |  |  | BHK | (Emara and Brinton, 2007) | |
|  |  |  |  |  | Mouse hepatitis, Coronavirus |  |  |  |  | LR7 | (Raaben et al., 2007) | |
|  |  |  |  |  | Poliovirus |  |  |  |  | HeLa | (White et al., 2007) | |
|  |  |  |  |  | Mammalian ortho-reovirus (MRV) |  |  |  |  | CV-1, DU-145, HeLa, COS7 | (Qin et al., 2009) | |
|  |  |  |  |  | Vesicular stomatitis virus 5h |  |  |  |  | HeLa | (Dinh et al., 2013) | |
|  |  |  |  |  | Hepatitis C virus |  |  |  |  | HuH7 | (Garaigorta et al., 2012) | |
|  |  |  |  |  |  |  | sorbitol |  |  | MTD-1, HeLa | (Fukuda et al., 2009; Sama et al., 2013) | |
|  |  |  |  | thapsigargin |  |  |  |  | (Dcp1) | NIH3T3, HEK293T | (Bosco et al., 2010; Loschi et al., 2009; Thomas et al., 2009) | |
|  |  |  |  |  |  |  |  | 5-FU |  | HeLa | (Kaehler et al., 2014) | |
|  |  |  |  |  |  |  |  | 5-fluoroudine |  | HeLa | (Kaehler et al., 2014) | |
|  |  |  |  |  |  |  |  | 5-azacytidine | (Dcp1) | HeLa | (Kaehler et al., 2014) | |
|  |  |  |  |  |  |  |  | 6-thioguanine | (Dcp1) | HeLa | (Kaehler et al., 2014) | |
|  |  |  |  |  |  |  |  | pateamine A |  | MEF | (Fujimura et al., 2012) | |
|  |  |  |  |  |  | selenite |  |  |  | U2OS | (Fujimura et al., 2012) | |
| TRAF2 | N |  | heat shock |  |  |  |  |  |  | HeLa | (Kim et al., 2008) | |
|  |  | sodium arsenite |  |  |  |  |  |  |  | HeLa | (Kim et al., 2008) | |
| TRN | N | sodium arsenite |  |  |  |  |  |  | Dcp1a, EDC4 (unstressed) | HeLa | (Chang and Tarn, 2009) | |
| TTP | Y |  |  |  |  |  |  | FCCP |  | DU145, HeLa, COS7 | (Stoecklin et al., 2004) | |
|  |  | sodium arsenite |  |  |  |  |  |  |  | DU145, HeLa, COS7 | (Chang and Tarn, 2009; Stoecklin et al., 2004) | |
|  |  |  | heat shock |  |  |  |  |  |  | NMuMG cells, COS7 | (Bann et al., 2014; Murata et al., 2005) | |
| Tudor-SN | Y |  | heat shock |  |  |  |  |  |  | HeLa | (Gao et al., 2010) | |
|  |  | sodium arsenite |  |  |  |  |  |  | (Dcp1) | HeLa | (Gao et al., 2010; Weissbach and Scadden, 2012) | |
| USP10 | N | sodium arsenite |  |  |  |  |  |  |  | HeLa, Jurkat, HEK293T, HuH7 | (Katoh et al., 2013; Takahashi et al., 2013) | |
| Upf1 | Y | sodium arsenite |  |  |  |  |  |  |  | HeLa, NFF | (Brown et al., 2011) | |
|  |  | hydrogen peroxide |  |  |  |  |  |  |  | HeLa | (Brown et al., 2011) | |
|  |  |  | heat shock |  |  |  |  |  |  | NFF | (Brown et al., 2011) | |
| Upf2 | Y |  | heat shock |  |  |  |  |  |  | NFF | (Brown et al., 2011) | |
| WDR62 | Y | sodium arsenite |  |  |  |  |  |  | (Dcp1) | HEK293T | (Wasserman et al., 2010) | |
|  |  |  | heat shock |  |  |  |  |  | (Dcp1) | HEK293T | (Wasserman et al., 2010) | |
| xrn1 | Y | sodium arsenite |  |  |  |  |  |  |  | DU145 | (Kedersha and Anderson, 2002) | |
| YB-1 | Y | sodium arsenite |  |  |  |  |  |  |  | HeLa, NG108-15, NRK | (Bounedjah et al., 2014; Chernov et al., 2009; Onishi et al., 2008; Tanaka et al., 2014) | |
|  |  |  | heat shock |  |  |  |  |  |  | HeLa, NMuMG, Sertoli cells | (Bann et al., 2014; ErLin et al., 2015; Suzuki et al., 2009) | |
| ZBP1 | Y | sodium arsenite |  |  |  |  |  |  | (Dcp1, Dcp2) | HeLa, U2OS | (Deigendesch et al., 2006; Stohr et al., 2006) | |
|  |  |  | heat shock |  |  |  |  |  |  | HeLa, U2OS | (Deigendesch et al., 2006; Stohr et al., 2006) | |

Andersson, M.K., A. Stahlberg, Y. Arvidsson, A. Olofsson, H. Semb, G. Stenman, O. Nilsson, and P. Aman. 2008. The multifunctional FUS, EWS and TAF15 proto-oncoproteins show cell type-specific expression patterns and involvement in cell spreading and stress response. *BMC Cell Biol*. 9:37.

Arimoto, K., H. Fukuda, S. Imajoh-Ohmi, H. Saito, and M. Takekawa. 2008. Formation of stress granules inhibits apoptosis by suppressing stress-responsive MAPK pathways. *Nat Cell Biol*. 10:1324-1332.

Ariumi, Y., M. Kuroki, Y. Kushima, K. Osugi, M. Hijikata, M. Maki, M. Ikeda, and N. Kato. 2011. Hepatitis C virus hijacks P-body and stress granule components around lipid droplets. *J Virol*. 85:6882-6892.

Athanasopoulos, V., A. Barker, D. Yu, A.H. Tan, M. Srivastava, N. Contreras, J. Wang, K.P. Lam, S.H. Brown, C.C. Goodnow, N.E. Dixon, P.J. Leedman, R. Saint, and C.G. Vinuesa. 2010. The ROQUIN family of proteins localizes to stress granules via the ROQ domain and binds target mRNAs. *FEBS J*. 277:2109-2127.

Aulas, A., S. Stabile, and C. Vande Velde. 2012. Endogenous TDP-43, but not FUS, contributes to stress granule assembly via G3BP. *Mol Neurodegener*. 7:54.

Baez, M.V., and G.L. Boccaccio. 2005. Mammalian Smaug is a translational repressor that forms cytoplasmic foci similar to stress granules. *J Biol Chem*. 280:43131-43140.

Baguet, A., S. Degot, N. Cougot, E. Bertrand, M.P. Chenard, C. Wendling, P. Kessler, H. Le Hir, M.C. Rio, and C. Tomasetto. 2007. The exon-junction-complex-component metastatic lymph node 51 functions in stress-granule assembly. *Journal of cell science*. 120:2774-2784.

Balzer, E., and E.G. Moss. 2007. Localization of the developmental timing regulator Lin28 to mRNP complexes, P-bodies and stress granules. *RNA biology*. 4:16-25.

Bann, D.V., A.R. Beyer, and L.J. Parent. 2014. A murine retrovirus co-Opts YB-1, a translational regulator and stress granule-associated protein, to facilitate virus assembly. *J Virol*. 88:4434-4450.

Bentmann, E., M. Neumann, S. Tahirovic, R. Rodde, D. Dormann, and C. Haass. 2012. Requirements for stress granule recruitment of fused in sarcoma (FUS) and TAR DNA-binding protein of 43 kDa (TDP-43). *J Biol Chem*. 287:23079-23094.

Blechingberg, J., Y. Luo, L. Bolund, C.K. Damgaard, and A.L. Nielsen. 2012. Gene expression responses to FUS, EWS, and TAF15 reduction and stress granule sequestration analyses identifies FET-protein non-redundant functions. *PLoS One*. 7:e46251.

Borghese, F., and T. Michiels. 2011. The leader protein of cardioviruses inhibits stress granule assembly. *J Virol*. 85:9614-9622.

Bosco, D.A., N. Lemay, H.K. Ko, H. Zhou, C. Burke, T.J. Kwiatkowski, Jr., P. Sapp, D. McKenna-Yasek, R.H. Brown, Jr., and L.J. Hayward. 2010. Mutant FUS proteins that cause amyotrophic lateral sclerosis incorporate into stress granules. *Human molecular genetics*. 19:4160-4175.

Bounedjah, O., B. Desforges, T.D. Wu, C. Pioche-Durieu, S. Marco, L. Hamon, P.A. Curmi, J.L. Guerquin-Kern, O. Pietrement, and D. Pastre. 2014. Free mRNA in excess upon polysome dissociation is a scaffold for protein multimerization to form stress granules. *Nucleic acids research*. 42:8678-8691.

Bravard, A., A. Campalans, M. Vacher, B. Gouget, C. Levalois, S. Chevillard, and J.P. Radicella. 2010. Inactivation by oxidation and recruitment into stress granules of hOGG1 but not APE1 in human cells exposed to sub-lethal concentrations of cadmium. *Mutat Res*. 685:61-69.

Brown, J.A., T.L. Roberts, R. Richards, R. Woods, G. Birrell, Y.C. Lim, S. Ohno, A. Yamashita, R.T. Abraham, N. Gueven, and M.F. Lavin. 2011. A novel role for hSMG-1 in stress granule formation. *Molecular and cellular biology*. 31:4417-4429.

Buchan, J.R., R.M. Kolaitis, J.P. Taylor, and R. Parker. 2013. Eukaryotic stress granules are cleared by autophagy and Cdc48/VCP function. *Cell*. 153:1461-1474.

Burry, R.W., and C.L. Smith. 2006. HuD distribution changes in response to heat shock but not neurotrophic stimulation. *J Histochem Cytochem*. 54:1129-1138.

Carpio, M.A., M.B. Decca, C. Lopez Sambrooks, E.S. Durand, G.G. Montich, and M.E. Hallak. 2013. Calreticulin-dimerization induced by post-translational arginylation is critical for stress granules scaffolding. *Int J Biochem Cell Biol*. 45:1223-1235.

Carpio, M.A., C. Lopez Sambrooks, E.S. Durand, and M.E. Hallak. 2010. The arginylation-dependent association of calreticulin with stress granules is regulated by calcium. *Biochem J*. 429:63-72.

Chalupnikova, K., S. Lattmann, N. Selak, F. Iwamoto, Y. Fujiki, and Y. Nagamine. 2008. Recruitment of the RNA helicase RHAU to stress granules via a unique RNA-binding domain. *J Biol Chem*. 283:35186-35198.

Chang, W.L., and W.Y. Tarn. 2009. A role for transportin in deposition of TTP to cytoplasmic RNA granules and mRNA decay. *Nucleic acids research*. 37:6600-6612.

Chernov, K.G., A. Barbet, L. Hamon, L.P. Ovchinnikov, P.A. Curmi, and D. Pastre. 2009. Role of microtubules in stress granule assembly: microtubule dynamical instability favors the formation of micrometric stress granules in cells. *J Biol Chem*. 284:36569-36580.

Chudinova, E.M., E.S. Nadezhdina, and P.A. Ivanov. 2012. Cellular acidosis inhibits assembly, disassembly, and motility of stress granules. *Biochemistry (Mosc)*. 77:1277-1284.

Colombrita, C., E. Zennaro, C. Fallini, M. Weber, A. Sommacal, E. Buratti, V. Silani, and A. Ratti. 2009. TDP-43 is recruited to stress granules in conditions of oxidative insult. *J Neurochem*. 111:1051-1061.

Courtney, S.C., S.V. Scherbik, B.M. Stockman, and M.A. Brinton. 2012. West nile virus infections suppress early viral RNA synthesis and avoid inducing the cell stress granule response. *J Virol*. 86:3647-3657.

Dang, Y., N. Kedersha, W.K. Low, D. Romo, M. Gorospe, R. Kaufman, P. Anderson, and J.O. Liu. 2006. Eukaryotic initiation factor 2alpha-independent pathway of stress granule induction by the natural product pateamine A. *J Biol Chem*. 281:32870-32878.

De Leeuw, F., T. Zhang, C. Wauquier, G. Huez, V. Kruys, and C. Gueydan. 2007. The cold-inducible RNA-binding protein migrates from the nucleus to cytoplasmic stress granules by a methylation-dependent mechanism and acts as a translational repressor. *Exp Cell Res*. 313:4130-4144.

Deigendesch, N., F. Koch-Nolte, and S. Rothenburg. 2006. ZBP1 subcellular localization and association with stress granules is controlled by its Z-DNA binding domains. *Nucleic acids research*. 34:5007-5020.

Dewey, C.M., B. Cenik, C.F. Sephton, D.R. Dries, P. Mayer, 3rd, S.K. Good, B.A. Johnson, J. Herz, and G. Yu. 2011. TDP-43 is directed to stress granules by sorbitol, a novel physiological osmotic and oxidative stressor. *Molecular and cellular biology*. 31:1098-1108.

Didiot, M.C., M. Subramanian, E. Flatter, J.L. Mandel, and H. Moine. 2009. Cells lacking the fragile X mental retardation protein (FMRP) have normal RISC activity but exhibit altered stress granule assembly. *Mol Biol Cell*. 20:428-437.

Dinh, P.X., L.K. Beura, P.B. Das, D. Panda, A. Das, and A.K. Pattnaik. 2013. Induction of stress granule-like structures in vesicular stomatitis virus-infected cells. *J Virol*. 87:372-383.

Eisinger-Mathason, T.S., J. Andrade, A.L. Groehler, D.E. Clark, T.L. Muratore-Schroeder, L. Pasic, J.A. Smith, J. Shabanowitz, D.F. Hunt, I.G. Macara, and D.A. Lannigan. 2008. Codependent functions of RSK2 and the apoptosis-promoting factor TIA-1 in stress granule assembly and cell survival. *Mol Cell*. 31:722-736.

Emara, M.M., and M.A. Brinton. 2007. Interaction of TIA-1/TIAR with West Nile and dengue virus products in infected cells interferes with stress granule formation and processing body assembly. *Proc Natl Acad Sci U S A*. 104:9041-9046.

Emara, M.M., K. Fujimura, D. Sciaranghella, V. Ivanova, P. Ivanov, and P. Anderson. 2012. Hydrogen peroxide induces stress granule formation independent of eIF2alpha phosphorylation. *Biochemical and biophysical research communications*. 423:763-769.

Emara, M.M., P. Ivanov, T. Hickman, N. Dawra, S. Tisdale, N. Kedersha, G.F. Hu, and P. Anderson. 2010. Angiogenin-induced tRNA-derived stress-induced RNAs promote stress-induced stress granule assembly. *J Biol Chem*. 285:10959-10968.

ErLin, S., W. WenJie, W. LiNing, L. BingXin, L. MingDe, S. Yan, and H. RuiFa. 2015. Musashi-1 maintains blood-testis barrier structure during spermatogenesis and regulates stress granule formation upon heat stress. *Mol Biol Cell*. 26:1947-1956.

Farny, N.G., N.L. Kedersha, and P.A. Silver. 2009. Metazoan stress granule assembly is mediated by P-eIF2alpha-dependent and -independent mechanisms. *Rna*. 15:1814-1821.

Figley, M.D., G. Bieri, R.M. Kolaitis, J.P. Taylor, and A.D. Gitler. 2014. Profilin 1 associates with stress granules and ALS-linked mutations alter stress granule dynamics. *J Neurosci*. 34:8083-8097.

Finnen, R.L., K.R. Pangka, and B.W. Banfield. 2012. Herpes simplex virus 2 infection impacts stress granule accumulation. *J Virol*. 86:8119-8130.

Fitzgerald, K.D., and B.L. Semler. 2013. Poliovirus infection induces the co-localization of cellular protein SRp20 with TIA-1, a cytoplasmic stress granule protein. *Virus Res*. 176:223-231.

Fournier, M.J., L. Coudert, S. Mellaoui, P. Adjibade, C. Gareau, M.F. Cote, N. Sonenberg, R.C. Gaudreault, and R. Mazroui. 2013. Inactivation of the mTORC1-eukaryotic translation initiation factor 4E pathway alters stress granule formation. *Molecular and cellular biology*. 33:2285-2301.

Fournier, M.J., C. Gareau, and R. Mazroui. 2010. The chemotherapeutic agent bortezomib induces the formation of stress granules. *Cancer Cell Int*. 10:12.

Fros, J.J., N.E. Domeradzka, J. Baggen, C. Geertsema, J. Flipse, J.M. Vlak, and G.P. Pijlman. 2012. Chikungunya virus nsP3 blocks stress granule assembly by recruitment of G3BP into cytoplasmic foci. *J Virol*. 86:10873-10879.

Fujimura, K., F. Kano, and M. Murata. 2008. Dual localization of the RNA binding protein CUGBP-1 to stress granule and perinucleolar compartment. *Exp Cell Res*. 314:543-553.

Fujimura, K., J. Katahira, F. Kano, Y. Yoneda, and M. Murata. 2009. Microscopic dissection of the process of stress granule assembly. *Biochim Biophys Acta*. 1793:1728-1737.

Fujimura, K., A.T. Sasaki, and P. Anderson. 2012. Selenite targets eIF4E-binding protein-1 to inhibit translation initiation and induce the assembly of non-canonical stress granules. *Nucleic acids research*. 40:8099-8110.

Fujimura, K., T. Suzuki, Y. Yasuda, M. Murata, J. Katahira, and Y. Yoneda. 2010. Identification of importin alpha1 as a novel constituent of RNA stress granules. *Biochim Biophys Acta*. 1803:865-871.

Fukuda, T., T. Naiki, M. Saito, and K. Irie. 2009. hnRNP K interacts with RNA binding motif protein 42 and functions in the maintenance of cellular ATP level during stress conditions. *Genes Cells*. 14:113-128.

Fung, G., C.S. Ng, J. Zhang, J. Shi, J. Wong, P. Piesik, L. Han, F. Chu, J. Jagdeo, E. Jan, T. Fujita, and H. Luo. 2013. Production of a dominant-negative fragment due to G3BP1 cleavage contributes to the disruption of mitochondria-associated protective stress granules during CVB3 infection. *PLoS One*. 8:e79546.

Gallois-Montbrun, S., B. Kramer, C.M. Swanson, H. Byers, S. Lynham, M. Ward, and M.H. Malim. 2007. Antiviral protein APOBEC3G localizes to ribonucleoprotein complexes found in P bodies and stress granules. *J Virol*. 81:2165-2178.

Gao, X., L. Ge, J. Shao, C. Su, H. Zhao, J. Saarikettu, X. Yao, Z. Yao, O. Silvennoinen, and J. Yang. 2010. Tudor-SN interacts with and co-localizes with G3BP in stress granules under stress conditions. *FEBS letters*. 584:3525-3532.

Garaigorta, U., M.H. Heim, B. Boyd, S. Wieland, and F.V. Chisari. 2012. Hepatitis C virus (HCV) induces formation of stress granules whose proteins regulate HCV RNA replication and virus assembly and egress. *J Virol*. 86:11043-11056.

Gareau, C., M.J. Fournier, C. Filion, L. Coudert, D. Martel, Y. Labelle, and R. Mazroui. 2011. p21(WAF1/CIP1) upregulation through the stress granule-associated protein CUGBP1 confers resistance to bortezomib-mediated apoptosis. *PLoS One*. 6:e20254.

Goodier, J.L., L. Zhang, M.R. Vetter, and H.H. Kazazian, Jr. 2007. LINE-1 ORF1 protein localizes in stress granules with other RNA-binding proteins, including components of RNA interference RNA-induced silencing complex. *Molecular and cellular biology*. 27:6469-6483.

Goulet, I., S. Boisvenue, S. Mokas, R. Mazroui, and J. Cote. 2008. TDRD3, a novel Tudor domain-containing protein, localizes to cytoplasmic stress granules. *Human molecular genetics*. 17:3055-3074.

Guil, S., J.C. Long, and J.F. Caceres. 2006. hnRNP A1 relocalization to the stress granules reflects a role in the stress response. *Molecular and cellular biology*. 26:5744-5758.

Hanley, L.L., D.R. McGivern, M.N. Teng, R. Djang, P.L. Collins, and R. Fearns. 2010. Roles of the respiratory syncytial virus trailer region: effects of mutations on genome production and stress granule formation. *Virology*. 406:241-252.

Henao-Mejia, J., Y. Liu, I.W. Park, J. Zhang, J. Sanford, and J.J. He. 2009. Suppression of HIV-1 Nef translation by Sam68 mutant-induced stress granules and nef mRNA sequestration. *Mol Cell*. 33:87-96.

Hinton, S.D., M.P. Myers, V.R. Roggero, L.A. Allison, and N.K. Tonks. 2010. The pseudophosphatase MK-STYX interacts with G3BP and decreases stress granule formation. *Biochem J*. 427:349-357.

Hofmann, I., M. Casella, M. Schnolzer, T. Schlechter, H. Spring, and W.W. Franke. 2006. Identification of the junctional plaque protein plakophilin 3 in cytoplasmic particles containing RNA-binding proteins and the recruitment of plakophilins 1 and 3 to stress granules. *Mol Biol Cell*. 17:1388-1398.

Hofmann, S., V. Cherkasova, P. Bankhead, B. Bukau, and G. Stoecklin. 2012. Translation suppression promotes stress granule formation and cell survival in response to cold shock. *Mol Biol Cell*. 23:3786-3800.

Hu, S., E.C. Claud, M.W. Musch, and E.B. Chang. 2010. Stress granule formation mediates the inhibition of colonic Hsp70 translation by interferon-gamma and tumor necrosis factor-alpha. *Am J Physiol Gastrointest Liver Physiol*. 298:G481-492.

Hua, Y., and J. Zhou. 2004. Survival motor neuron protein facilitates assembly of stress granules. *FEBS letters*. 572:69-74.

Kaehler, C., J. Isensee, T. Hucho, H. Lehrach, and S. Krobitsch. 2014. 5-Fluorouracil affects assembly of stress granules based on RNA incorporation. *Nucleic acids research*. 42:6436-6447.

Katoh, H., T. Okamoto, T. Fukuhara, H. Kambara, E. Morita, Y. Mori, W. Kamitani, and Y. Matsuura. 2013. Japanese encephalitis virus core protein inhibits stress granule formation through an interaction with Caprin-1 and facilitates viral propagation. *J Virol*. 87:489-502.

Kedersha, N., and P. Anderson. 2002. Stress granules: sites of mRNA triage that regulate mRNA stability and translatability. *Biochemical Society transactions*. 30:963-969.

Kedersha, N., S. Chen, N. Gilks, W. Li, I.J. Miller, J. Stahl, and P. Anderson. 2002. Evidence that ternary complex (eIF2-GTP-tRNA(i)(Met))-deficient preinitiation complexes are core constituents of mammalian stress granules. *Mol Biol Cell*. 13:195-210.

Kedersha, N., G. Stoecklin, M. Ayodele, P. Yacono, J. Lykke-Andersen, M.J. Fritzler, D. Scheuner, R.J. Kaufman, D.E. Golan, and P. Anderson. 2005. Stress granules and processing bodies are dynamically linked sites of mRNP remodeling. *The Journal of cell biology*. 169:871-884.

Kedersha, N.L., M. Gupta, W. Li, I. Miller, and P. Anderson. 1999. RNA-binding proteins TIA-1 and TIAR link the phosphorylation of eIF-2 alpha to the assembly of mammalian stress granules. *The Journal of cell biology*. 147:1431-1442.

Khong, A., and E. Jan. 2011. Modulation of stress granules and P bodies during dicistrovirus infection. *J Virol*. 85:1439-1451.

Kim, B., H.J. Cooke, and K. Rhee. 2012. DAZL is essential for stress granule formation implicated in germ cell survival upon heat stress. *Development*. 139:568-578.

Kim, J.E., I. Ryu, W.J. Kim, O.K. Song, J. Ryu, M.Y. Kwon, J.H. Kim, and S.K. Jang. 2008. Proline-rich transcript in brain protein induces stress granule formation. *Molecular and cellular biology*. 28:803-813.

Kimball, S.R., R.L. Horetsky, D. Ron, L.S. Jefferson, and H.P. Harding. 2003. Mammalian stress granules represent sites of accumulation of stalled translation initiation complexes. *Am J Physiol Cell Physiol*. 284:C273-284.

Kobayashi, T., S. Winslow, L. Sunesson, U. Hellman, and C. Larsson. 2012. PKCalpha binds G3BP2 and regulates stress granule formation following cellular stress. *PLoS One*. 7:e35820.

Kolobova, E., A. Efimov, I. Kaverina, A.K. Rishi, J.W. Schrader, A.J. Ham, M.C. Larocca, and J.R. Goldenring. 2009. Microtubule-dependent association of AKAP350A and CCAR1 with RNA stress granules. *Exp Cell Res*. 315:542-555.

Kunde, S.A., L. Musante, A. Grimme, U. Fischer, E. Muller, E.E. Wanker, and V.M. Kalscheuer. 2011. The X-chromosome-linked intellectual disability protein PQBP1 is a component of neuronal RNA granules and regulates the appearance of stress granules. *Human molecular genetics*. 20:4916-4931.

Kwon, S., Y. Zhang, and P. Matthias. 2007. The deacetylase HDAC6 is a novel critical component of stress granules involved in the stress response. *Genes Dev*. 21:3381-3394.

Langereis, M.A., Q. Feng, and F.J. van Kuppeveld. 2013. MDA5 localizes to stress granules, but this localization is not required for the induction of type I interferon. *J Virol*. 87:6314-6325.

Lavut, A., and D. Raveh. 2012. Sequestration of highly expressed mRNAs in cytoplasmic granules, P-bodies, and stress granules enhances cell viability. *PLoS genetics*. 8:e1002527.

Lee, Y.J., H.M. Wei, L.Y. Chen, and C. Li. 2014. Localization of SERBP1 in stress granules and nucleoli. *FEBS J*. 281:352-364.

Leung, A.K., J.M. Calabrese, and P.A. Sharp. 2006. Quantitative analysis of Argonaute protein reveals microRNA-dependent localization to stress granules. *Proc Natl Acad Sci U S A*. 103:18125-18130.

Li, C.H., T. Ohn, P. Ivanov, S. Tisdale, and P. Anderson. 2010. eIF5A promotes translation elongation, polysome disassembly and stress granule assembly. *PLoS One*. 5:e9942.

Lian, X.J., and I.E. Gallouzi. 2009. Oxidative Stress Increases the Number of Stress Granules in Senescent Cells and Triggers a Rapid Decrease in p21waf1/cip1 Translation. *J Biol Chem*. 284:8877-8887.

Lin, J.C., M. Hsu, and W.Y. Tarn. 2007. Cell stress modulates the function of splicing regulatory protein RBM4 in translation control. *Proc Natl Acad Sci U S A*. 104:2235-2240.

Linder, B., O. Plottner, M. Kroiss, E. Hartmann, B. Laggerbauer, G. Meister, E. Keidel, and U. Fischer. 2008. Tdrd3 is a novel stress granule-associated protein interacting with the Fragile-X syndrome protein FMRP. *Human molecular genetics*. 17:3236-3246.

Lindquist, M.E., A.W. Lifland, T.J. Utley, P.J. Santangelo, and J.E. Crowe, Jr. 2010. Respiratory syncytial virus induces host RNA stress granules to facilitate viral replication. *J Virol*. 84:12274-12284.

Lindsay, A.J., and M.W. McCaffrey. 2011. Myosin Va is required for P body but not stress granule formation. *J Biol Chem*. 286:11519-11528.

Linero, F.N., M.G. Thomas, G.L. Boccaccio, and L.A. Scolaro. 2011. Junin virus infection impairs stress-granule formation in Vero cells treated with arsenite via inhibition of eIF2alpha phosphorylation. *J Gen Virol*. 92:2889-2899.

Liu-Yesucevitz, L., A. Bilgutay, Y.J. Zhang, T. Vanderweyde, A. Citro, T. Mehta, N. Zaarur, A. McKee, R. Bowser, M. Sherman, L. Petrucelli, and B. Wolozin. 2010. Tar DNA binding protein-43 (TDP-43) associates with stress granules: analysis of cultured cells and pathological brain tissue. *PLoS One*. 5:e13250.

Loschi, M., C.C. Leishman, N. Berardone, and G.L. Boccaccio. 2009. Dynein and kinesin regulate stress-granule and P-body dynamics. *Journal of cell science*. 122:3973-3982.

Mahboubi, H., E. Seganathy, D. Kong, and U. Stochaj. 2013. Identification of Novel Stress Granule Components That Are Involved in Nuclear Transport. *PLoS One*. 8:e68356.

Matsuki, H., M. Takahashi, M. Higuchi, G.N. Makokha, M. Oie, and M. Fujii. 2013. Both G3BP1 and G3BP2 contribute to stress granule formation. *Genes Cells*. 18:135-146.

Mazroui, R., S. Di Marco, R.J. Kaufman, and I.E. Gallouzi. 2007. Inhibition of the ubiquitin-proteasome system induces stress granule formation. *Mol Biol Cell*. 18:2603-2618.

Mazroui, R., M.E. Huot, S. Tremblay, C. Filion, Y. Labelle, and E.W. Khandjian. 2002. Trapping of messenger RNA by Fragile X Mental Retardation protein into cytoplasmic granules induces translation repression. *Human molecular genetics*. 11:3007-3017.

Mazroui, R., R. Sukarieh, M.E. Bordeleau, R.J. Kaufman, P. Northcote, J. Tanaka, I. Gallouzi, and J. Pelletier. 2006. Inhibition of ribosome recruitment induces stress granule formation independently of eukaryotic initiation factor 2alpha phosphorylation. *Mol Biol Cell*. 17:4212-4219.

McDonald, K.K., A. Aulas, L. Destroismaisons, S. Pickles, E. Beleac, W. Camu, G.A. Rouleau, and C. Vande Velde. 2011. TAR DNA-binding protein 43 (TDP-43) regulates stress granule dynamics via differential regulation of G3BP and TIA-1. *Human molecular genetics*. 20:1400-1410.

McInerney, G.M., N.L. Kedersha, R.J. Kaufman, P. Anderson, and P. Liljestrom. 2005. Importance of eIF2alpha phosphorylation and stress granule assembly in alphavirus translation regulation. *Mol Biol Cell*. 16:3753-3763.

Mok, B.W., W. Song, P. Wang, H. Tai, Y. Chen, M. Zheng, X. Wen, S.Y. Lau, W.L. Wu, K. Matsumoto, K.Y. Yuen, and H. Chen. 2012. The NS1 protein of influenza A virus interacts with cellular processing bodies and stress granules through RNA-associated protein 55 (RAP55) during virus infection. *J Virol*. 86:12695-12707.

Mokas, S., J.R. Mills, C. Garreau, M.J. Fournier, F. Robert, P. Arya, R.J. Kaufman, J. Pelletier, and R. Mazroui. 2009. Uncoupling stress granule assembly and translation initiation inhibition. *Mol Biol Cell*. 20:2673-2683.

Murata, T., N. Morita, K. Hikita, K. Kiuchi, K. Kiuchi, and N. Kaneda. 2005. Recruitment of mRNA-destabilizing protein TIS11 to stress granules is mediated by its zinc finger domain. *Exp Cell Res*. 303:287-299.

Nadezhdina, E.S., A.J. Lomakin, A.A. Shpilman, E.M. Chudinova, and P.A. Ivanov. 2010. Microtubules govern stress granule mobility and dynamics. *Biochim Biophys Acta*. 1803:361-371.

Ng, S.K., R. Weissbach, G.E. Ronson, and A.D. Scadden. 2013. Proteins that contain a functional Z-DNA-binding domain localize to cytoplasmic stress granules. *Nucleic acids research*. 41:9786-9799.

Nikpour, P., M.E. Baygi, C. Steinhoff, C. Hader, A.C. Luca, S.J. Mowla, and W.A. Schulz. 2011. The RNA binding protein Musashi1 regulates apoptosis, gene expression and stress granule formation in urothelial carcinoma cells. *J Cell Mol Med*. 15:1210-1224.

Nonhoff, U., M. Ralser, F. Welzel, I. Piccini, D. Balzereit, M.L. Yaspo, H. Lehrach, and S. Krobitsch. 2007. Ataxin-2 interacts with the DEAD/H-box RNA helicase DDX6 and interferes with P-bodies and stress granules. *Mol Biol Cell*. 18:1385-1396.

Ohn, T., N. Kedersha, T. Hickman, S. Tisdale, and P. Anderson. 2008. A functional RNAi screen links O-GlcNAc modification of ribosomal proteins to stress granule and processing body assembly. *Nat Cell Biol*. 10:1224-1231.

Okonski, K.M., and C.E. Samuel. 2013. Stress granule formation induced by measles virus is protein kinase PKR dependent and impaired by RNA adenosine deaminase ADAR1. *J Virol*. 87:756-766.

Onishi, H., Y. Kino, T. Morita, E. Futai, N. Sasagawa, and S. Ishiura. 2008. MBNL1 associates with YB-1 in cytoplasmic stress granules. *J Neurosci Res*. 86:1994-2002.

Onomoto, K., M. Jogi, J.S. Yoo, R. Narita, S. Morimoto, A. Takemura, S. Sambhara, A. Kawaguchi, S. Osari, K. Nagata, T. Matsumiya, H. Namiki, M. Yoneyama, and T. Fujita. 2012. Critical role of an antiviral stress granule containing RIG-I and PKR in viral detection and innate immunity. *PLoS One*. 7:e43031.

Panas, M.D., M. Varjak, A. Lulla, K.E. Eng, A. Merits, G.B. Karlsson Hedestam, and G.M. McInerney. 2012. Sequestration of G3BP coupled with efficient translation inhibits stress granules in Semliki Forest virus infection. *Mol Biol Cell*. 23:4701-4712.

Pare, J.M., N. Tahbaz, J. Lopez-Orozco, P. LaPointe, P. Lasko, and T.C. Hobman. 2009. Hsp90 regulates the function of argonaute 2 and its recruitment to stress granules and P-bodies. *Mol Biol Cell*. 20:3273-3284.

Parker, S.J., J. Meyerowitz, J.L. James, J.R. Liddell, P.J. Crouch, K.M. Kanninen, and A.R. White. 2012. Endogenous TDP-43 localized to stress granules can subsequently form protein aggregates. *Neurochem Int*. 60:415-424.

Piotrowska, J., S.J. Hansen, N. Park, K. Jamka, P. Sarnow, and K.E. Gustin. 2010. Stable formation of compositionally unique stress granules in virus-infected cells. *J Virol*. 84:3654-3665.

Pizzo, E., C. Sarcinelli, J. Sheng, S. Fusco, F. Formiggini, P. Netti, W. Yu, G. D'Alessio, and G.F. Hu. 2013. Ribonuclease/angiogenin inhibitor 1 regulates stress-induced subcellular localization of angiogenin to control growth and survival. *Journal of cell science*. 126:4308-4319.

Pothof, J., N.S. Verkaik, I.W. van, E.A. Wiemer, V.T. Ta, G.T. van der Horst, N.G. Jaspers, D.C. van Gent, J.H. Hoeijmakers, and S.P. Persengiev. 2009. MicroRNA-mediated gene silencing modulates the UV-induced DNA-damage response. *EMBO J*. 28:2090-2099.

Qi, D., S. Huang, R. Miao, Z.G. She, T. Quinn, Y. Chang, J. Liu, D. Fan, Y.E. Chen, and M. Fu. 2011. Monocyte chemotactic protein-induced protein 1 (MCPIP1) suppresses stress granule formation and determines apoptosis under stress. *J Biol Chem*. 286:41692-41700.

Qin, Q., C. Hastings, and C.L. Miller. 2009. Mammalian orthoreovirus particles induce and are recruited into stress granules at early times postinfection. *J Virol*. 83:11090-11101.

Quaresma, A.J., G.C. Bressan, L.M. Gava, D.C. Lanza, C.H. Ramos, and J. Kobarg. 2009. Human hnRNP Q re-localizes to cytoplasmic granules upon PMA, thapsigargin, arsenite and heat-shock treatments. *Exp Cell Res*. 315:968-980.

Raaben, M., M.J. Groot Koerkamp, P.J. Rottier, and C.A. de Haan. 2007. Mouse hepatitis coronavirus replication induces host translational shutoff and mRNA decay, with concomitant formation of stress granules and processing bodies. *Cell Microbiol*. 9:2218-2229.

Rojas, M., G.W. Farr, C.F. Fernandez, L. Lauden, J.C. McCormack, and S.L. Wolin. 2012. Yeast Gis2 and its human ortholog CNBP are novel components of stress-induced RNP granules. *PLoS One*. 7:e52824.

Ruggieri, A., E. Dazert, P. Metz, S. Hofmann, J.P. Bergeest, J. Mazur, P. Bankhead, M.S. Hiet, S. Kallis, G. Alvisi, C.E. Samuel, V. Lohmann, L. Kaderali, K. Rohr, M. Frese, G. Stoecklin, and R. Bartenschlager. 2012. Dynamic oscillation of translation and stress granule formation mark the cellular response to virus infection. *Cell Host Microbe*. 12:71-85.

Sahoo, P.K., P. Murawala, P.T. Sawale, M.R. Sahoo, M.M. Tripathi, S.R. Gaikwad, V. Seshadri, and J. Joseph. 2012. Wnt signalling antagonizes stress granule assembly through a Dishevelled-dependent mechanism. *Biol Open*. 1:109-119.

Sama, R.R., C.L. Ward, L.J. Kaushansky, N. Lemay, S. Ishigaki, F. Urano, and D.A. Bosco. 2013. FUS/TLS assembles into stress granules and is a prosurvival factor during hyperosmolar stress. *J Cell Physiol*. 228:2222-2231.

Schwartz, J.C., C.C. Ebmeier, E.R. Podell, J. Heimiller, D.J. Taatjes, and T.R. Cech. 2012. FUS binds the CTD of RNA polymerase II and regulates its phosphorylation at Ser2. *Genes Dev*. 26:2690-2695.

Seguin, S.J., F.F. Morelli, J. Vinet, D. Amore, S. De Biasi, A. Poletti, D.C. Rubinsztein, and S. Carra. 2014. Inhibition of autophagy, lysosome and VCP function impairs stress granule assembly. *Cell Death Differ*.

Shih, J.W., W.T. Wang, T.Y. Tsai, C.Y. Kuo, H.K. Li, and Y.H. Wu Lee. 2012. Critical roles of RNA helicase DDX3 and its interactions with eIF4E/PABP1 in stress granule assembly and stress response. *Biochem J*. 441:119-129.

Singleton, R.S., P. Liu-Yi, F. Formenti, W. Ge, R. Sekirnik, R. Fischer, J. Adam, P.J. Pollard, A. Wolf, A. Thalhammer, C. Loenarz, E. Flashman, A. Yamamoto, M.L. Coleman, B.M. Kessler, P. Wappner, C.J. Schofield, P.J. Ratcliffe, and M.E. Cockman. 2014. OGFOD1 catalyzes prolyl hydroxylation of RPS23 and is involved in translation control and stress granule formation. *Proc Natl Acad Sci U S A*. 111:4031-4036.

Solomon, S., Y. Xu, B. Wang, M.D. David, P. Schubert, D. Kennedy, and J.W. Schrader. 2007. Distinct structural features of caprin-1 mediate its interaction with G3BP-1 and its induction of phosphorylation of eukaryotic translation initiation factor 2alpha, entry to cytoplasmic stress granules, and selective interaction with a subset of mRNAs. *Molecular and cellular biology*. 27:2324-2342.

Souquere, S., S. Mollet, M. Kress, F. Dautry, G. Pierron, and D. Weil. 2009. Unravelling the ultrastructure of stress granules and associated P-bodies in human cells. *Journal of cell science*. 122:3619-3626.

Stoecklin, G., T. Stubbs, N. Kedersha, S. Wax, W.F. Rigby, T.K. Blackwell, and P. Anderson. 2004. MK2-induced tristetraprolin:14-3-3 complexes prevent stress granule association and ARE-mRNA decay. *EMBO J*. 23:1313-1324.

Stohr, N., M. Lederer, C. Reinke, S. Meyer, M. Hatzfeld, R.H. Singer, and S. Huttelmaier. 2006. ZBP1 regulates mRNA stability during cellular stress. *The Journal of cell biology*. 175:527-534.

Sukarieh, R., N. Sonenberg, and J. Pelletier. 2009. The eIF4E-binding proteins are modifiers of cytoplasmic eIF4E relocalization during the heat shock response. *Am J Physiol Cell Physiol*. 296:C1207-1217.

Suzuki, Y., M. Minami, M. Suzuki, K. Abe, S. Zenno, M. Tsujimoto, K. Matsumoto, and Y. Minami. 2009. The Hsp90 inhibitor geldanamycin abrogates colocalization of eIF4E and eIF4E-transporter into stress granules and association of eIF4E with eIF4G. *J Biol Chem*. 284:35597-35604.

Takahashi, M., M. Higuchi, H. Matsuki, M. Yoshita, T. Ohsawa, M. Oie, and M. Fujii. 2013. Stress granules inhibit apoptosis by reducing reactive oxygen species production. *Molecular and cellular biology*. 33:815-829.

Tanaka, T., S. Ohashi, and S. Kobayashi. 2014. Roles of YB-1 under arsenite-induced stress: translational activation of HSP70 mRNA and control of the number of stress granules. *Biochim Biophys Acta*. 1840:985-992.

Thomas, M.G., L.J. Martinez Tosar, M.A. Desbats, C.C. Leishman, and G.L. Boccaccio. 2009. Mammalian Staufen 1 is recruited to stress granules and impairs their assembly. *Journal of cell science*. 122:563-573.

Thomas, M.G., L.J. Martinez Tosar, M. Loschi, J.M. Pasquini, J. Correale, S. Kindler, and G.L. Boccaccio. 2005. Staufen recruitment into stress granules does not affect early mRNA transport in oligodendrocytes. *Mol Biol Cell*. 16:405-420.

Tourriere, H., K. Chebli, L. Zekri, B. Courselaud, J.M. Blanchard, E. Bertrand, and J. Tazi. 2003. The RasGAP-associated endoribonuclease G3BP assembles stress granules. *The Journal of cell biology*. 160:823-831.

Tsai, N.P., P.C. Ho, and L.N. Wei. 2008. Regulation of stress granule dynamics by Grb7 and FAK signalling pathway. *EMBO J*. 27:715-726.

Unsworth, H., S. Raguz, H.J. Edwards, C.F. Higgins, and E. Yague. 2010. mRNA escape from stress granule sequestration is dictated by localization to the endoplasmic reticulum. *FASEB J*. 24:3370-3380.

Wang, Y., G. Lacroix, J. Haines, E. Doukhanine, G. Almazan, and S. Richard. 2010. The QKI-6 RNA binding protein localizes with the MBP mRNAs in stress granules of glial cells. *PLoS One*. 5.

Wasserman, T., K. Katsenelson, S. Daniliuc, T. Hasin, M. Choder, and A. Aronheim. 2010. A novel c-Jun N-terminal kinase (JNK)-binding protein WDR62 is recruited to stress granules and mediates a nonclassical JNK activation. *Mol Biol Cell*. 21:117-130.

Wehner, K.A., S. Schutz, and P. Sarnow. 2010. OGFOD1, a novel modulator of eukaryotic translation initiation factor 2alpha phosphorylation and the cellular response to stress. *Molecular and cellular biology*. 30:2006-2016.

Weissbach, R., and A.D. Scadden. 2012. Tudor-SN and ADAR1 are components of cytoplasmic stress granules. *Rna*. 18:462-471.

Wen, X., X. Huang, B.W. Mok, Y. Chen, M. Zheng, S.Y. Lau, P. Wang, W. Song, D.Y. Jin, K.Y. Yuen, and H. Chen. 2014. NF90 exerts antiviral activity through regulation of PKR phosphorylation and stress granules in infected cells. *J Immunol*. 192:3753-3764.

White, J.P., A.M. Cardenas, W.E. Marissen, and R.E. Lloyd. 2007. Inhibition of cytoplasmic mRNA stress granule formation by a viral proteinase. *Cell Host Microbe*. 2:295-305.

White, J.P., and R.E. Lloyd. 2011. Poliovirus unlinks TIA1 aggregation and mRNA stress granule formation. *J Virol*. 85:12442-12454.

Wilczynska, A., C. Aigueperse, M. Kress, F. Dautry, and D. Weil. 2005. The translational regulator CPEB1 provides a link between dcp1 bodies and stress granules. *Journal of cell science*. 118:981-992.

Wippich, F., B. Bodenmiller, M.G. Trajkovska, S. Wanka, R. Aebersold, and L. Pelkmans. 2013. Dual specificity kinase DYRK3 couples stress granule condensation/dissolution to mTORC1 signaling. *Cell*. 152:791-805.

Yamaguchi, A., and K. Kitajo. 2012. The effect of PRMT1-mediated arginine methylation on the subcellular localization, stress granules, and detergent-insoluble aggregates of FUS/TLS. *PLoS One*. 7:e49267.

Yu, J.H., W.H. Yang, T. Gulick, K.D. Bloch, and D.B. Bloch. 2005. Ge-1 is a central component of the mammalian cytoplasmic mRNA processing body. *Rna*. 11:1795-1802.

Zhang, J., K. Okabe, T. Tani, and T. Funatsu. 2011. Dynamic association-dissociation and harboring of endogenous mRNAs in stress granules. *Journal of cell science*. 124:4087-4095.

Zhang, P., Y. Li, J. Xia, J. He, J. Pu, J. Xie, S. Wu, L. Feng, X. Huang, and P. Zhang. 2014. IPS-1 plays an essential role in dsRNA-induced stress granule formation by interacting with PKR and promoting its activation. *Journal of cell science*. 127:2471-2482.

Zhu, C.H., J. Kim, J.W. Shay, and W.E. Wright. 2008. SGNP: an essential Stress Granule/Nucleolar Protein potentially involved in 5.8s rRNA processing/transport. *PLoS One*. 3:e3716.
